# Prognostic and Immunological Role of Cuproptosis-Related Gene MTF1 in Pan-Cancer

**DOI:** 10.7150/jca.98749

**Published:** 2024-09-09

**Authors:** Chao Zhang, Sifen Wang, Hailin Tang, Renchun Lai, Qiaoting Cai, Yaorong Su, Hao Wu, Yongwen Huang

**Affiliations:** 1 State Key Laboratory of Oncology in South China, Guangdong Provincial Clinical Research Center for Cancer, Sun Yat-Sen University Cancer Center, Guangzhou. China.; 2 Jiangmen Central Hospital, Jiangmen 529030, China.

**Keywords:** metal regulatory transcription factor 1, prognosis, tumor immune microenvironment, pan-cancer, cuproptosis

## Abstract

Metal regulatory transcription factor 1 (MTF1) has been reported to induce the expression of metallothionein and other genes involved in metal homeostasis. However, the role of MTF1 in pan-cancer and tumor immunity remains unclear. In this study, we conducted a series of bioinformatics analyses to investigate the clinical significance and potential functions of MTF1 across various types of cancer. By employing bioinformatics algorithms and immunofluorescence assays, we analyzed the associations between MTF1 and immune infiltration in the tumor microenvironment as well as the expression levels of immune-related molecules. Our findings revealed dysregulation of MTF1 in pan-cancer along with its correlation with certain clinicopathological features, suggesting its diagnostic and prognostic value for multiple cancer types. Furthermore, our immune-associated analyses and assays demonstrated strong correlations between MTF1 expression and plasmacytoid dendritic cells (pDC), central memory T cells (Tcm), as well as several immune biomarkers. Subsequent in vitro assays indicated that MTF1 reduced the sensitivity of cancer cells to cuproptosis. Overall, our study highlights that MTF1 may serve as a promising biomarker for prognosis assessment and a potential therapeutic target for more effective treatment strategies against various cancers.

## Introduction

Despite advances in diagnosis and treatment in recent years, the incidence and mortality of various malignant tumors are still on the rise, which is a major health problem worldwide 1, 2. As one of the most basic components of living organisms, metal elements are widely involved in various biological processes of biological cells, such as cell proliferation, migration, transport, metabolism, and death 3. It plays a key role in the regulation of various stages of life activities through a variety of complex mechanisms by influencing the structure and function of enzymes, for example 4. With the participation of multiple groups of molecules, their intake and metabolism are regulated in a complex and precise manner to ensure body homeostasis 5. It is worth noting that the involvement of metal ions in the functional regulation of a great number of immune cells and immune molecules in the body has been widely reported in recent years 6-8. Therefore, the study of upstream and downstream regulation of metal ions may have positive significance for the prediction of cancer immunity and therapy efficacy.

MTF1 encodes a widely expressed transcription factor that binds metal response elements (MRE) in the promoters of heavy-metal response genes and regulates their transcription and expression. Previous studies have confirmed that zinc could bind MTF1 and induce its translocation to the nucleus, triggering the expression of downstream genes 9. Two years ago, a new mechanism of regulatory cell death, named cuproptosis, was discovered by scientists. They found that the direct binding of copper to the lipoylated components of the tricarboxylic acid (TCA) cycle resulted in the aggregation of lipoylated proteins and the subsequent loss of iron-sulfur clusters, which led to proteotoxic stress and cell death 10-12. Moreover, cuproptosis is a novel therapeutic target for cancer 11, 13. In addition, the screening results suggested that MTF1 might be a resistance factor to cuproptosis 14. Given the critical role of regulatory cell death in cancer treatment resistance 15, the relationship between MTF1 and cuproptosis and the role of MTF1 in cancer and immunity need to be further clarified.

In the present study, the expression profiles and clinical significance of MTF1 in pan-cancer were revealed. Subsequently, we speculated the potential function of MTF1 in tumors by constructing a protein interaction network and a series of enrichment analyses. In addition, we analyzed the association between MTF1 expression and immune subtypes, immune cell infiltration, and immune-related biomarkers in a variety of malignant tumors. Through some experiments, we explored the effect of MTF1 knockdown on cuproptosis in tumor cells. Therefore, this study revealed the comprehensive role of MTF1 in pan-cancer and provided a potential predictor for immune infiltration and expression of immune-related biomarkers in some types of cancers.

## Methods

### Expression Data Acquisition and Processing

The RNA expression data and the Genotype-Tissue Expression (GTEx) data of 33 kinds of tumor and normal tissues were obtained from The Cancer Genome Atlas (TCGA) database (https://portal.gdc.cancer.gov/) and the University of California Santa Cruz (UCSC Xena) (https://xena.ucsc.edu/), which are two free data portal of large-scale cancer genome projects. The downloaded data were transformed from FPKM (fragments per kilobase per million) to TPM (transcripts per million reads) and then a log_2_(TPM+1) conversion was performed. The analysis of MTF1 mRNA expression between cancer and paired or non-paired normal samples, and the visualization of the results were achieved using R software (version 3.6.3) and the ggplot2 package (version 3.3.3).

### Diagnostic and Prognostic Roles of MTF1 in Pan-Cancer

We used the receiver operating characteristic (ROC) curve to estimate the diagnostic efficacy of MTF1 for tumor versus normal in different cancer types. The analysis was conducted using the pROC package (version 1.17.0.1) and the area under curve (AUC) above 0.8 was considered to have a good diagnostic performance. As for the prognostic role, we obtained the clinical prognosis data of pan-cancer in TCGA database. We compared three common survival indicators including the overall survival (OS), the progression-free survival (PFS), and the disease-specific survival (DSS) of patients with high and low MTF1 expression (percentiles 0-50 vs. 50-100) by drawing the Kaplan-Meier plot using the R software and survminer package (version 0.4.9). The results of the above analyses were integrated and shown through a radar map and forest maps.

### Cox Regression Analyses and Nomogram Models Construction

Through a Venn diagram, we screened out two cancer types with significant correlation between all prognostic indicators and MTF1 expression, which were kidney renal clear cell carcinoma (KIRC) and brain lower grade glioma (LGG). To further explore the role of MTF1 in patients with KIRC and LGG, we used the univariate Cox regression analysis to calculate the association between the expression level of MTF1 and the OS of the patients in the two cohorts. Along with other clinicopathological features, variables that were significant in the univariate Cox regression analyses (*P* < 0.05) were included in the multivariate Cox regression analyses. The result was statistically significant in multivariate Cox regression analysis when the *P*-value was less than 0.05. In addition, two nomogram models for predicting the prognosis of KIRC and LGG were constructed by including the variables with statistical significance in the univariate Cox regression analyses, and the predictive efficacy was tested by the C-index, prognostic calibration analysis, and time-dependent ROC curve. These analyses were performed using the survival package (version 3.2-10) and the rms package (version 6.2-0) in R software.

### Genetic and Epigenetic Alterations in Pan-Cancer

To explore the potential reason for MTF1 expression dysregulation in pan-cancer, copy number variation (CNV) and methylation analyses of the MTF1 gene were performed using the gene set cancer analysis (GSCA) website (http://bioinfo.life.hust.edu.cn/GSCA/#/) 16. We analyzed and demonstrated the differences in MTF1 methylation in different cancer species and the correlation between methylation and expression, as well as the profiles of MTF1 CNV and its correlation with gene expression. The relationship between methylation, CNV status, and the survival of patients was also assessed. More detailed CpG methylation site data of MTF1 in KIRC, LGG, head and neck squamous cell carcinoma (HNSC), and lung squamous cell carcinoma (LUSC) was downloaded from the MethSurv database (https://biit.cs.ut.ee/methsurv/) 17. The results were presented using the ggplot2 package through lollipop charts, pie charts, and radar charts. As for the analysis of the genetic mutation profiles of MTF1, we referred to TCGA Pan-Cancer Atlas Studies (including 10967 samples of 32 studies) on an online database cBioPortal (https://www.cbioportal.org/) 18, 19.

### Drug Sensitivity Prediction

Drug response data from the Genomics of Drug Sensitivity in Cancer (GDSC) database 20 and the Cancer Therapeutics Response Portal (CTRP) data was also obtained from the GSCA website. The Spearman correlation was calculated between drug sensitivity and MTF1 expression. Positive correlation indicated that tumor cells highly expressing MTF1 were prone to resistance to drugs while negative correlation meant the opposite.

### Protein-Protein Interaction (PPI) Analysis

The Search Tool for the Retrieval of Interacting Genes/Proteins (STRING) database (https://string-db.org/), which hosts a large collection of integrated and consolidated PPI data was used to obtain PPI network information for MTF1 21. The interactors including compounds could be found in the search tool for interacting chemicals (STITCH) database (http://stitch.embl.de/) 22. We chose all the interaction sources and set the minimum required interaction score at 0.4 in using both databases. The downloaded analysis results were processed and then visualized using the Cytoscape software (version 3.9.1).

### Gene Ontology (GO) and Kyoto Encyclopedia of Genes and Genomes (KEGG) Analysis

To obtain a gene set associated with MTF1, we drew a Venn diagram to find the common interactors of MTF1 obtained from the STRING database and the STITCH database. Then, the GO and KEGG pathway analyses were conducted with the cluster-Profiler package (version 3.14.3) in R software 23. The enrichment results of biological process (BP), cellular component (CC), molecular function (MF), and KEGG were sorted in the order of rising p-value, from which the top several results were shown in the bubble chart.

### Gene Set Enrichment Analysis (GSEA) in Pan-Cancer

GSEA is a computational method that determines whether a previously defined gene set shows statistically significant differences between two biological states 24. Firstly, we grouped the gene expression matrix in KIRC, LGG, LUSC, and HNSC according to the level of MTF1 expression. We made analysis materials according to the regulations and selected the MSigDB Collection (C2.all.v7.2.symbols.GMT) for analysis. The results were screened with a false discovery rate (FDR) < 0.05 and adjusted *P* < 0.05 cut-offs. The screened items were then sorted by the normalized enrichment score (NES) from highest to lowest and we showed the top five results for each cancer species.

### Single-Cell Analysis of MTF1

The online database CancerSea (http://biocc.hrbmu.edu.cn/CancerSEA/) is a platform providing gene expression data and functional status of cancer cells at the single-cell level 30. The correlations between MTF1 expression and 14 kinds of tumor-associated functional status including invasion, metastasis, proliferation, epithelial-mesenchymal transition (EMT), angiogenesis, apoptosis, cell cycle, differentiation, DNA damage, DNA repair, hypoxia, inflammation, quiescence, and stemness were explored in 18 cancers. The threshold for a significant correlation was set at 0.3 and p-value less than 0.05. A new approach, the t-distributed stochastic neighbor embedding (t-SNE), was used to identify the expression distribution of MTF1 in cancer 25.

### Immune Subtype Analyses in Pan-Cancer

To investigate the relationship between MTF1 expression and immune subtypes of different cancers, we made use of the "Subtype" module of the TISIDB database (http://cis.hku.hk/TISIDB/index.php/) 26. The immune subtypes of tumors can be divided into the following six groups according to their molecular characteristics: C1 (wound healing), C2 (interferon-γ (IFN-γ) dominant), C3 (inflammatory), C4 (lymphocyte depleted), C5 (immunologically quiet), and C6 (transforming growth factor-β (TGF-β) dominant) 27. We compared the expression of MTF1 in pan-cancer to see if there were differences among different immune subtypes.

### Immune Infiltration Analyses in Pan-Cancer

To find out the relationship between MTF1 and immune infiltration status in pan-cancer, we analyzed 24 kinds of immune cells including activated dendritic cells (aDC), B cells, CD8+ T cells, cytotoxic cells, dendritic cells (DC), eosinophils, immature dendritic cells (iDC), macrophages, mast cells, neutrophils, CD56bright natural killer (NK) cells, CD56dim NK cells, NK cells, plasmacytoid dendritic cells (pDC), T cells, T helper cells, central memory T cells (Tcm), effector memory T cells (Tem), follicular helper T cells (Tfh), gamma delta T cells, Th17cells, Th2 cells and regulatory T cells (Treg) by calculating the Spearman correlation using the ssGSEA (single sample gene set enrichment analysis) algorithm of the GSVA package (Version 1.34.0) in R software 28, 29. The threshold for a significant correlation was set at 0.3 and *P*-value less than 0.05.

### Immune Associated Biomarkers Analysi90es in Pan-Cancer

Immune-associated molecules play an important role in achieving the complicated function properly of the tumor microenvironment 30. We obtained immune-associated gene sets that included 37 immune stimulators, 22 immunosuppressors, 21 major histocompatibility complexes (MHCs), and 41 chemokines by referring to the TISIDB. We explored the Spearman correlation between expression of MTF1 and immune stimulators, immunosuppressors, MHCs, and chemokines in pan-cancer. Results were shown by heatmaps and correlation coefficients greater than 0.3 and *P*-values less than 0.05 were considered statistically significant.

### Multiple Fluorescence Immunohistochemistry

We obtained tumor tissue sections from the tissue specimen bank of Sun Yat-Sen University Cancer Center and the ethics were approved. We stained samples via a multiplex fluorescence immunohistochemical kit, PDOne four-color TSA-RM-275 (20 T) (cat 10001100020, Panovue, China) according to the manual provided. Paraffin-embedded samples were sequentially incubated with primary antibodies and horseradish peroxidase (HRP)-conjugated secondary antibodies. Then, tyrosine signal amplification (TSA) was performed to label antigens, after each TSA labeling step, we removed the primary and secondary antibodies through a microwave treatment for heat-induced antigen retrieval. After the sample was eluted, the next antigen was labeled, and this procedure was repeated for all three markers. According to previous studies, BDCA2 and CD62L were selected as molecular markers indicating pDC and Tcm infiltration, respectively 38, 39. Anti-MTF1 (dilution 1:200, 25383-1-AP, Proteintech, USA), anti-BDCA2 (dilution 1:200, ab240557, Abcam, UK), and anti-CD62L (dilution 1:200, ab244495, Abcam, UK) were used as primary antibodies. The dyes Opal520, Opal570 and 4′-6′-diamidino-2-phenylindole (DAPI, Sigma-Aldrich, USA) were used for staining. Fluorescence-positive cells were evaluated by Caseviewer (CV 2.3) and Pannoramic Viewer (PV 1.15.3) software.

### Cell Lines and Culture Conditions

Two chosen human cell lines which expressed MTF1 moderately, MDA-MB-231 and Kyse30, were obtained from the State Key Laboratory of Oncology in South China (Sun Yat-Sen University Cancer Center, China). These cells were cultured in Dulbecco's Modified Eagle Medium (DMEM) with 10% fetal bovine serum (FBS) at 37 ℃ in a 5% CO2 environment.

### Knockdown of MTF1 Using a Small Interfering RNA (siRNA)

Two siRNAs targeting MTF1 and a nonspecific scrambled siRNA sequence were purchased (Kidan Biosciences Co., Ltd., China). Lipofectamine 3000 reagent (Invitrogen, USA) was used to transfect the siRNAs into cells according to the manufacturer's instructions. The efficacy of the siRNAs was verified using western blotting. The sequences of the siRNAs were as follows:

MTF1 si-1, 5'-AGGUGAUUAUUGAGUCUUGUATT-3', MTF1 si-2, 5'-UCCGUGCUGUAAGCCUUUGUUTT-3'.

### RNA Isolation and Quantitative Real-Time PCR (qRT-PCR)

Total RNA was harvested using the TRIzol reagent (15596026, Invitrogen, USA) and reverse transcribed using the kit from EZBioscience (A0010CGQ, EZBioscience, USA). The resulting cDNA was subjected to qRT-PCR analysis, performed in triplicate, using the 2×SYBR Green qPCR Master Mix (A0001-R2, EZBioscience, USA) on a CFX96 Real-Time System C1000 Cycler (Bio-Rad Laboratories, USA). The experiment was performed in strict accordance with the user instructions and expression of MTF1 was calculated with the 2^-ΔΔCt^ method and normalized to the glyceraldehyde-3-phosphate dehydrogenase (GAPDH). The primer sequences used in the PCR reactions were listed as follows:

MTF1, forward GAGGCTTCACACAGGGAAAACG, reverse GCTTTTCCACAGCCATCGTGATC; GAPDH, forward GTCTCCTCTGACTTCAACAGCG, reverse ACCACCCTGTTGCTGTAGCCAA.

### Western Blotting

Total cellular proteins were extracted using radioimmunoprecipitation assay (RIPA) lysis buffer (Thermo Fisher Scientific, USA) and quantified by using a Bio-Rad DC protein assay kit II (Bio-Rad, USA), separated by electrophoresis on 10% sodium dodecyl sulfate-polyacrylamide gel (SDS-PAGE) gels and electrotransferred onto a Hybond ECL transfer membrane (Amersham Pharmacia, Piscataway, USA). The membranes were blocked with 5% non-fat milk and incubated with the appropriate primary antibodies. The membranes were subsequently incubated with labeled secondary antibodies and detected with enhanced chemiluminescence reagents (Thermo Fisher Scientific, USA). The immunoreactive membranes were visualized by an ECL chemiluminescent substrate reagent kit (Thermo Fisher Scientific, USA). The antibodies and their respective dilutions were as follows: anti-MTF1 (dilution 1:1000, 25383-1-AP, Proteintech, USA), anti-caspase3 (dilution 1:1000, 9662, Cell Signaling Technology, USA), anti-cleaved-caspase3 (dilution 1:800, 9664, Cell Signaling Technology, USA), GAPDH (dilution 1:1000, 5174, Cell Signaling Technology, USA).

### Colony Formation Assay

Cells in the logarithmic growth stage were taken, digested with trypsin, and blown into single cells, and 500 cells/well were inoculated in each group in a 6-well culture plate. After culture on day 14, an appropriate amount of Giemsa dye was applied after methanol fixation for 30 minutes, and then photographs were taken by the imager and counted by ImageJ.

### Cell Viability Assay

After digestion in the exponential growth phase, MDA-MB-231 and Kyse30 cells were seeded in 96-well plates (5000 cells/well) and treated with corresponding processes. At the indicated time point, namely 24 hours after processing with drugs including different concentrations of Elesclomol-CuCl2 (1:1) and paclitaxel, 10ul CCK8 solution was added into the wells and incubated with cells according to the protocol of the kit. Then the Thermomax microplate reader was used to measure the absorbance of each well at a wavelength of 450 nm (A450).

### Statistical Analysis

R software (version 3.6.3) was used to conduct statistical analysis. Differences between groups were compared using the Wilcoxon rank-sum test, one-way ANOVA test, or Student's t-test, as appropriate. The Spearman coefficients were calculated between variables and the threshold for a significant correlation was set at 0.3. Data were reported as statistics and 95% confidence intervals (CIs). Differences were considered statistically significant when *P* < 0.05.

## Results

### MTF1 Expression Analysis in Pan-Cancer

**Figure 1:**
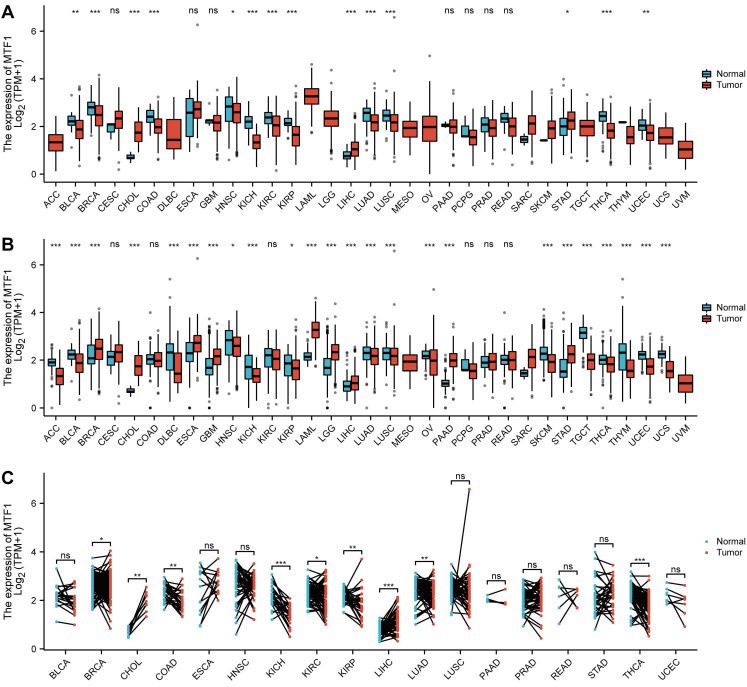
Expression profiles of MTF1 in pan-cancer. (**A**) The mRNA expression level of MTF1 in pan-cancer from TCGA database. (**B**) The mRNA expression level of MTF1 from the GTEx database. (**C**) The mRNA expression level of MTF1 in paired tumor and normal tissues in various cancers. * *P* < 0.05, ** *P* < 0.01, *** *P* < 0.001.

To explore the expression of MTF1, we analyzed the mRNA expression data of 33 kinds of cancers from the GTEx and TCGA databases. MTF1 was differentially expressed in most cancer types including adrenocortical carcinoma (ACC), bladder urothelial carcinoma (BLCA), breast invasive carcinoma (BRCA), cholangiocarcinoma (CHOL), diffuse large B-cell lymphoma (DLBC), esophageal carcinoma (ESCA), glioblastoma multiforme (GBM), HNSC, kidney chromophobe (KICH), kidney renal papillary cell carcinoma (KIRP), acute myeloid leukemia (LAML), LGG, liver hepatocellular carcinoma (LIHC), lung adenocarcinoma (LUAD), LUSC, ovarian serous cystadenocarcinoma (OV), pancreatic adenocarcinoma (PAAD), skin cutaneous melanoma (SKCM), stomach adenocarcinoma (STAD), testicular germ cell tumors (TGCT), thyroid carcinoma (THCA), thymoma (THYM), uterine corpus endometrial carcinoma (UCEC) and uterine carcinosarcoma (UCS). Upregulation of MTF1 was found in BRCA, CHOL, ESCA, GBM, LAML, LGG, LIHC, PAAD, and STAD, while downregulation of MTF1 was found in ACC, BLCA, DLBC, HNSC, KICH, KIRP, LUAD, LUSC, OV, SKCM, TGCT, THCA, THYM, UCEC, and UCS (**Fig. 1A, 1B**). To verify the above results, we also analyzed the expression of MTF1 in tumor tissues and its paired normal tissues in various cancers. The result showed that the expression of MTF1 was significantly higher in CHOL and LIHC while lower in BRCA, colon adenocarcinoma (COAD), KICH, KIRC, KIRP, LUAD, and THCA (**Fig. 1C**).

### Association Between MTF1 and Clinicopathological Features

**Figure 2:**
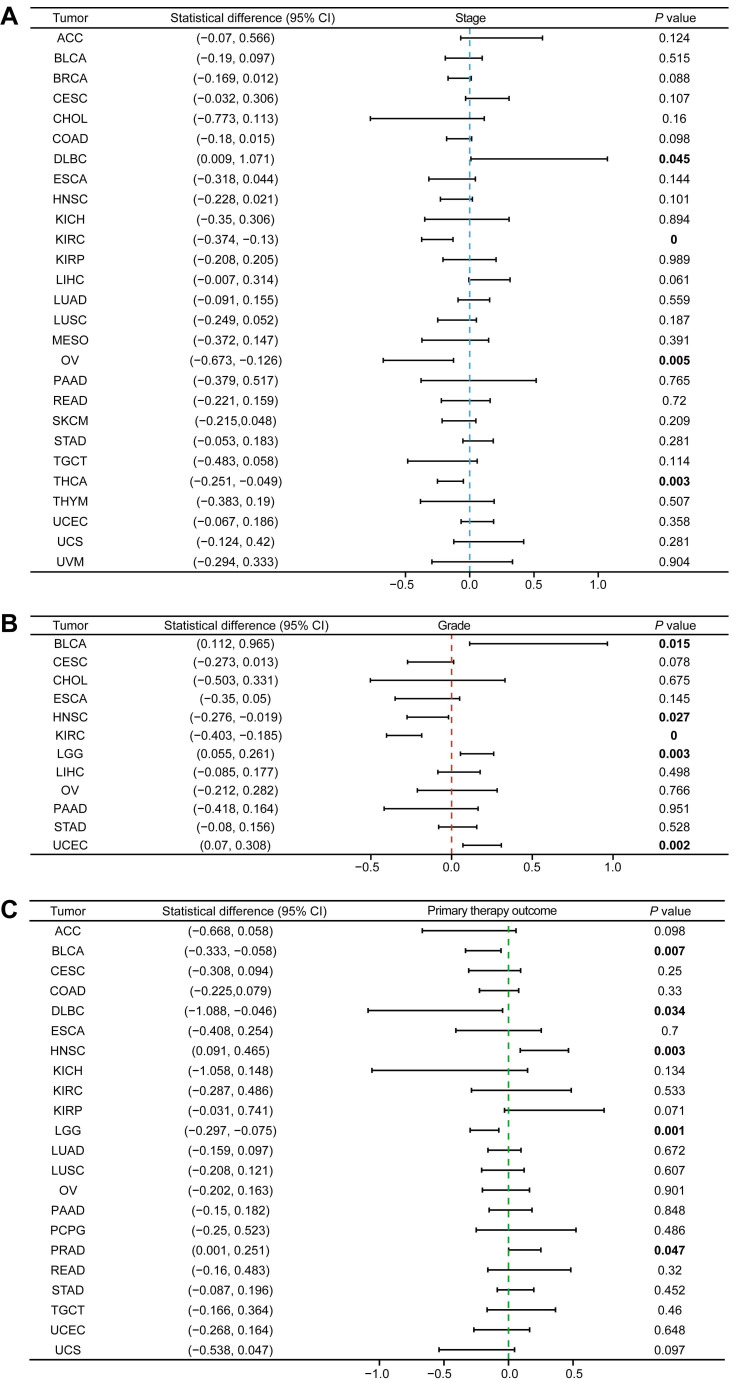
Expression of MTF1 was correlated with clinicopathological features in pan-cancer. Correlation between MTF1 expression and pathological stage (**A**), histological grade (**B**), and primary therapy outcome (**C**). Bold *P*-value indicates statistical significance.

Since the role of MTF1 in pan-cancer has not been clarified, we analyzed the correlation between the expression of MTF1 and pathological stage, histologic grade, and primary therapy outcome. We found that higher expression of MTF1 was related to higher stages in DLBC, and lower stages in KIRC, OV, and THCA (**Fig. 2A, Supplementary Fig. S1A**). Higher expression as a risk factor for worse histological grade was found in BLCA, LGG, and UCEC, and as a protective factor in HNSC and KIRC (**Fig. 2B, Supplementary Fig. S1B**). As for the primary therapy outcome, complete response (CR) patients had lower MTF1 expression than non-CR patients in BLCA, DLBC, and LGG, while in HNSC and prostate adenocarcinoma (PRAD), the results were reversed (**Fig. 2C, Supplementary Fig. S1C**).

### Survival Analysis of MTF1 in Pan-Cancer

**Figure 3:**
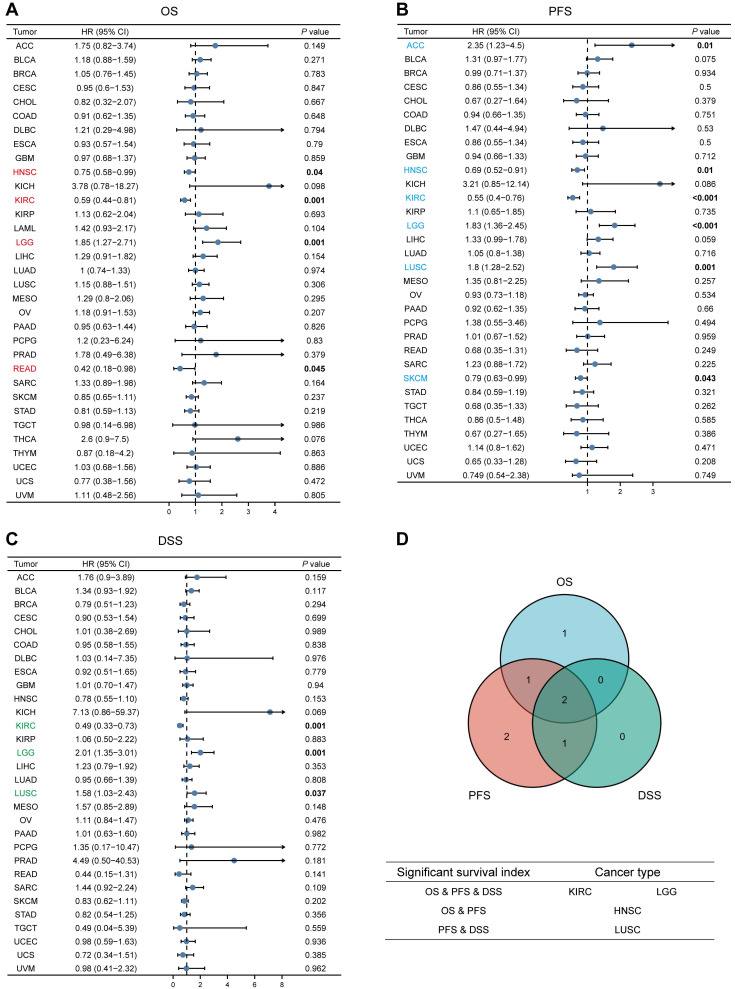
Survival analyses of MTF1 expression in pan-cancer. Relationship between MTF1 expression and OS (**A**), PFS (**B**), and DSS (**C**). (**D**) Venn plots of tumors with statistically significant survival indicators. Colored and bold font indicate statistical significance.

We analyzed the relationship between MTF1 expression and OS, PFS, and DSS in different types of cancer to evaluate its prognostic value. According to the results, patients with a higher expression of MTF1 had a shorter OS in LGG (hazard ratio (HR) = 1.85, *P* = 0.001) and a longer OS in HNSC (HR = 0.75, *P* = 0.04), KIRC (HR = 0.59, *P* = 0.001), and rectum adenocarcinoma (READ) (HR = 0.42, *P* = 0.045) (**Fig. 3A, Supplementary Fig. S2A**). High expression was significantly associated with poor PFS in ACC (HR = 2.35, *P* = 0.01), LGG (HR = 1.83, *P* < 0.001), and LUSC (HR = 1.8, *P* = 0.001), whereas a good prognosis was associated with high expression in HNSC (HR = 0.69, *P* = 0.01), KIRC (HR = 0.55, *P* < 0.001), and SKCM (HR = 0.79, *P* = 0.043) (**Fig. 3B, Supplementary Fig. S2B**). In addition, the relationship between MTF1 expression and DSS in KIRC (HR = 0.49, *P* = 0.001), LGG (HR = 2.01, *P* = 0.001), and LUSC (HR = 1.58, *P* = 0.037) was consistent with OS and PFS (**Fig. 3C, Supplementary Fig. S2C**).

### Univariate and Multivariate Cox Regression Analysis

**Figure 4:**
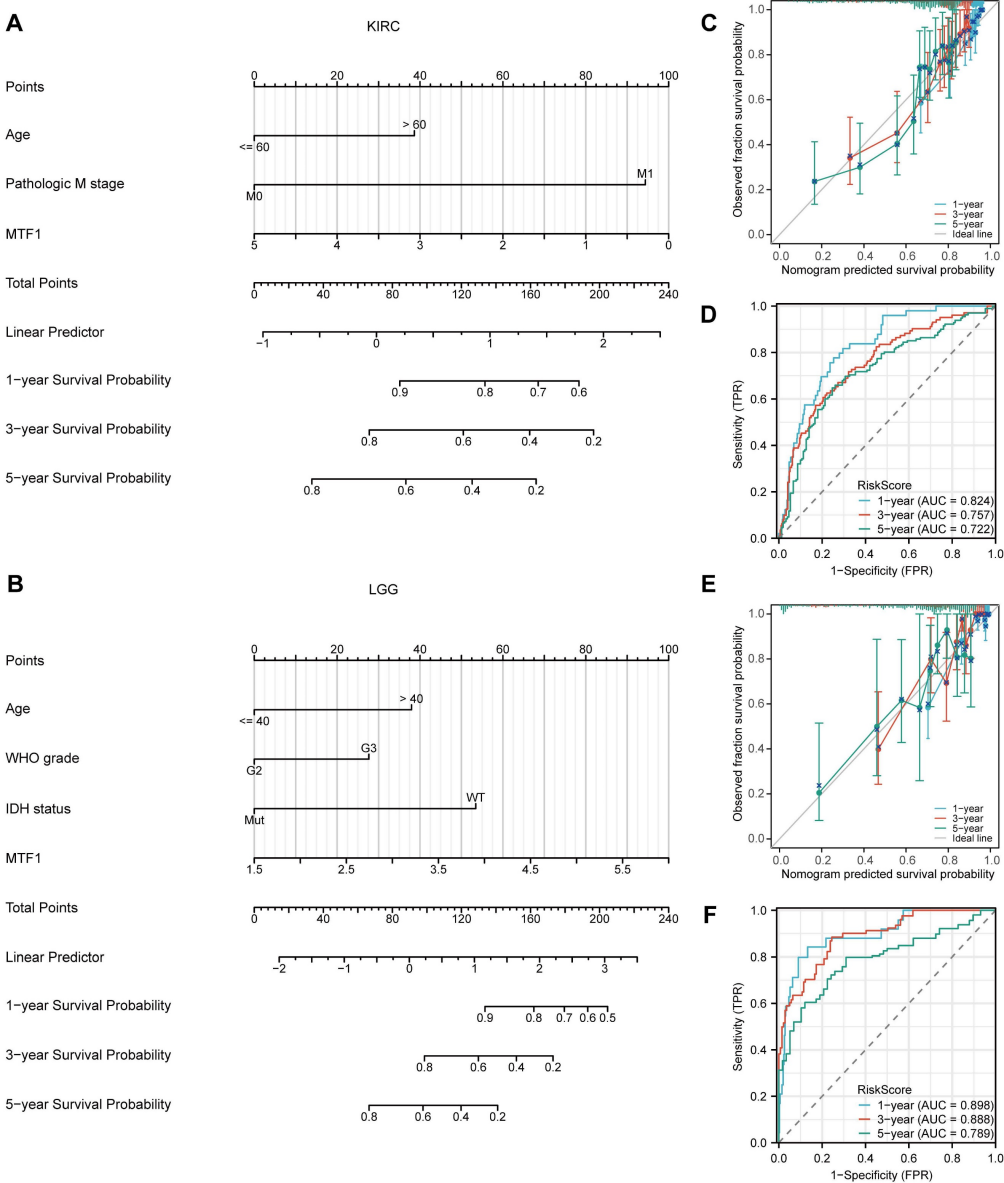
Prognostic roles of MTF1 in KIRC and LGG. (**A**) A nomogram predicting survival probability at 1, 3, and 5 years for KIRC. (**B**) A nomogram predicting survival probability for LGG. (**C**) Calibration analysis to test the prediction performance of the nomogram model in KIRC. (**D**) The time-dependent ROC curve was used to evaluate the predictive efficiency of MTF1 expression on KIRC. (**E**) Calibration analysis in LGG. (**F**) The time-dependent ROC curve of MTF1 expression in LGG.

We have found that there were significant differences in the expression levels of MTF1 in KIRC and LGG patients with different outcomes of the three survival indicators. Therefore, univariate and multivariate Cox regression analyses were performed to further understand the prognostic factors of KIRC and LGG. Baseline information for KIRC and LGG patients were presented separately (**Supplementary Table S1, S2**). The univariate Cox regression analysis showed that the factors that might influence the survival of patients with KIRC were age (*P* < 0.001), T stage (*P* < 0.001), N stage (*P* < 0.001), M stage (*P* < 0.001), pathological stage (*P* < 0.001), histologic grade (*P* < 0.001), and MTF1 expression (*P* < 0.001). Among them, age (*P* = 0.022), M stage (*P* < 0.001), and expression of MTF1 (*P* = 0.009) were independent prognostic factors for KIRC by multivariate regression analysis (**Table 1**). As for LGG, age (*P* < 0.001), WHO grade (*P* < 0.001), histological type (*P* < 0.05), isocitrate dehydrogenase (IDH) status (*P* < 0.001), and MTF1 expression (*P* < 0.001) were statistically significant in univariate Cox regression analysis, and age (*P* < 0.001), WHO grade (*P* = 0.002), IDH status (*P* < 0.001), and MTF1 expression (*P* = 0.005) were independent prognostic factors for LGG (**Table 2**). After obtaining the prognostic factors of KIRC and LGG, we constructed the prognostic prediction nomograms at 1, 3, and 5 years for them, respectively (**Fig. 4A, 4B**) (C-index of KIRC: 0.736, 95% CI: 0.717-0.755; C-index of LGG: 0.826, 95% CI: 0.804-0.847). The prognostic calibration analyses and time-dependent ROC curves supported the good predictive performance of the nomograms (all AUC > 0.7) (**Fig. 4C-F**).

### Diagnostic Value of MTF1 in Pan-Cancer

**Figure 5:**
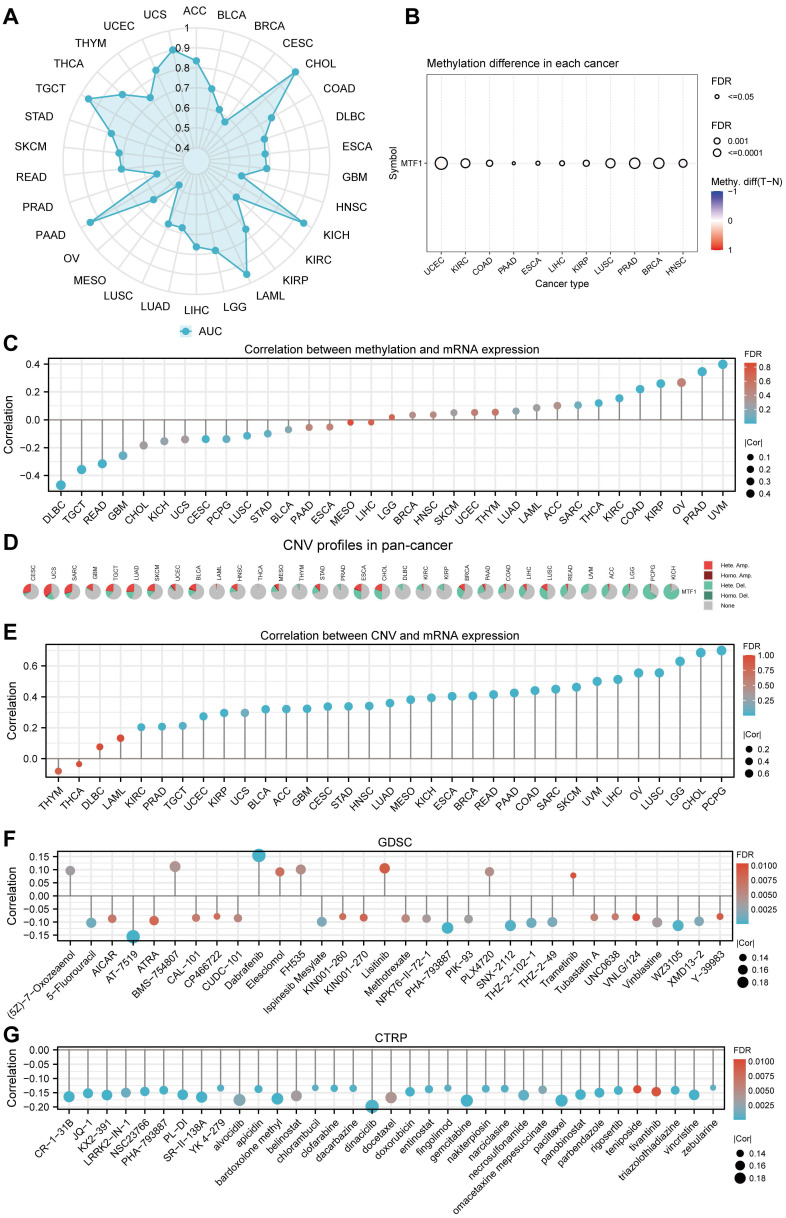
Diagnostic value, methylation, CNV, and drug sensitivity analyses of MTF1 in pan-cancer. (**A**) AUCs of the ROC curves across cancers are summarized in a radar chart. (**B**) Cancers in which methylation differs between cancer and normal controls. (**C**) Correlation between methylation and MTF1 expression in pan-cancer. (**D**) CNV profiles in pan-cancer. (**E**) Correlation between CNV and MTF1 expression in pan-cancer. (**F**) Correlation between MTF1 expression and drug sensitivity in GDSC. (**G**) Correlation between MTF1 expression and drug sensitivity in CTRP.

To explore the diagnostic value of MTF1 in pan-cancer, the ROC curves were drawn using the expression of MTF1 to distinguish the tumor from the corresponding normal tissue. We showed the results with a radar map and found that MTF1 expression had good diagnostic efficacy in ACC (AUC = 0.835), CHOL (AUC = 1), KICH (AUC = 0.953), LAML (AUC = 0.953), PAAD (AUC = 0.946), TGCT (AUC = 0.956), THCA (AUC = 0.831), UCEC (AUC = 0.831), and UCS (AUC = 0.902) (**Fig. 5A, Supplementary Fig. S3**).

### Methylation, CNV, and Drug Sensitivity Analysis

By analyzing the methylation and CNV of the MTF1 gene in pan-cancer, we may provide a potential explanation for the abnormal expression of MTF1 in different cancer species. Analysis results of GSCA online database suggested that there were significant differences between tumor and normal methylation modification in BRCA, COAD, ESCA, HNSC, KIRC, KIRP, LIHC, LUSC, PAAD, PRAD, and UCEC (all FDR < 0.01) (**Fig. 5B**). Further analyses revealed that methylation level was significantly negatively correlated with MTF1 expression in DLBC, READ, and TGCT, while positively correlated with MTF1 expression in PRAD and uveal melanoma (UVM) (|correlation| > 0.3 and FDR < 0.05) (**Fig. 5C**). By analyzing the pan-cancer methylation modification data in the MethSurv database, we found that abnormal methylation levels of some methylation sites in LAML, ACC, BLCA, BRCA, cervical squamous cell carcinoma and endocervical adenocarcinoma (CESC), COAD, GBM, HNSC, KICH, KIRC, KIRP, LGG, LIHC, LUAD, LUSC, mesothelioma (MESO), PAAD, READ, sarcoma (SARC), SKCM, STAD, UCEC, UCS, and UVM were significantly correlated with OS of cancer patients (all *P* < 0.05).

The methylation sites of MTF1 in KIRC, LGG, KICH, and LUSC are shown in forest maps (Supplementary Fig.S4). CNV profiles of pan-cancer including homozygous amplification, homozygous deletion, heterozygous amplification, and heterozygous deletion are shown in pie diagrams (**Fig. 5D, Supplementary Fig. S5A, S5B**). Further analysis showed that there was a significant positive correlation between CNV and MTF1 expression in most cancer species (|correlation| > 0.3 and FDR < 0.05) (**Fig. 5E**). Additional analysis results and the association between CNV and survival indicators including OS, PFS, and DSS in pan-cancer are presented in the supplementary materials (**Supplementary Fig. S5C-E**). Survival analysis also indicated that methylation status was related to survival indicators in BLCA, KIRC, LUAD, PRAD, LAML, and UVM (**Supplementary Fig. S5F-H**). Moreover, we demonstrated the relationship between MTF1 expression and the efficacy of some commonly used chemotherapy drugs by analyzing two drug sensitivity-related databases, GDSC and CTRP. High expression of MTF1 was correlated with high sensitivity to several compounds including 5-fluorouracil, vinblastine, doxorubicin, gemcitabine, paclitaxel, and so on (**Fig. 5F, 5G**).

### Genetic Alteration of MTF1 in Pan-Cancer

**Figure 6:**
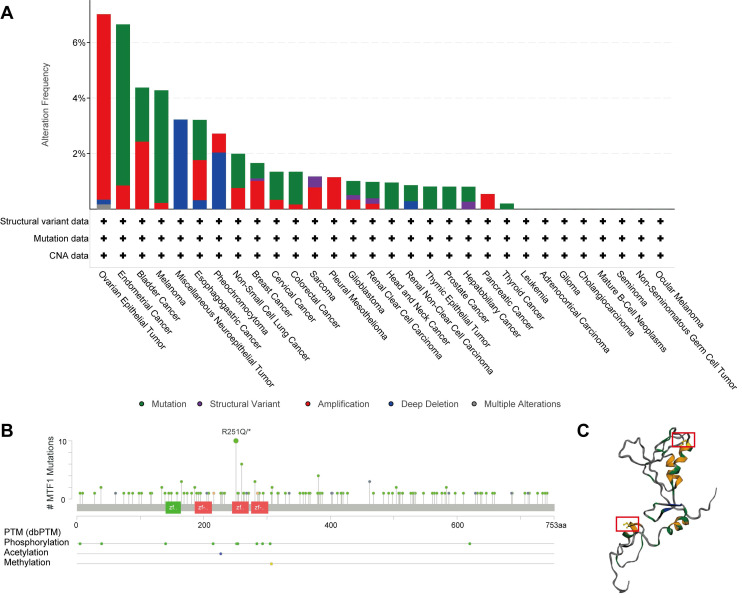
Genetic changes of MTF1 in pan-cancer. (**A**) Genetic alterations of MTF1 across cancers. (**B**) The mutation sites on the MTF1 gene. (**C**) 3D structural diagram of the position of the highest-frequency R251Q mutation on the MTF1 protein.

Besides abnormal gene expression, gene mutation and structural variation could be involved in the development of disease by affecting the structure and function of gene products. A total of 10,967 patients with various kinds of cancers from 32 studies in the cBioPortal database were analyzed. Mutations, structural variants, and CNV of the MTF1 gene were found in 22 out of 30 kinds of tumors. Among them, mutation and amplification were the most important genetic changes (**Fig. 6A**). As shown in the results, the frequency of MTF1 alteration was the highest in ovarian epithelial tumors at a rate of 7.02%, which also had the highest frequency of amplification (6.68%). Endometrial cancer had the highest incidence of mutation (5.8%) and miscellaneous neuroepithelial tumors had the uppermost rate of deletion (3.23%). Further analysis revealed that a total of 143 mutations were in the MTF1 gene, among which arginine to glutamine transition was the most common at position 251 located in the zinc finger double domain (245-271) (**Fig. 6B**). The change in spatial conformation caused by a mutation at this site is shown in the 3D structure diagram (**Fig. 6C**).

### PPI Analysis and Enrichment Analysis

**Figure 7:**
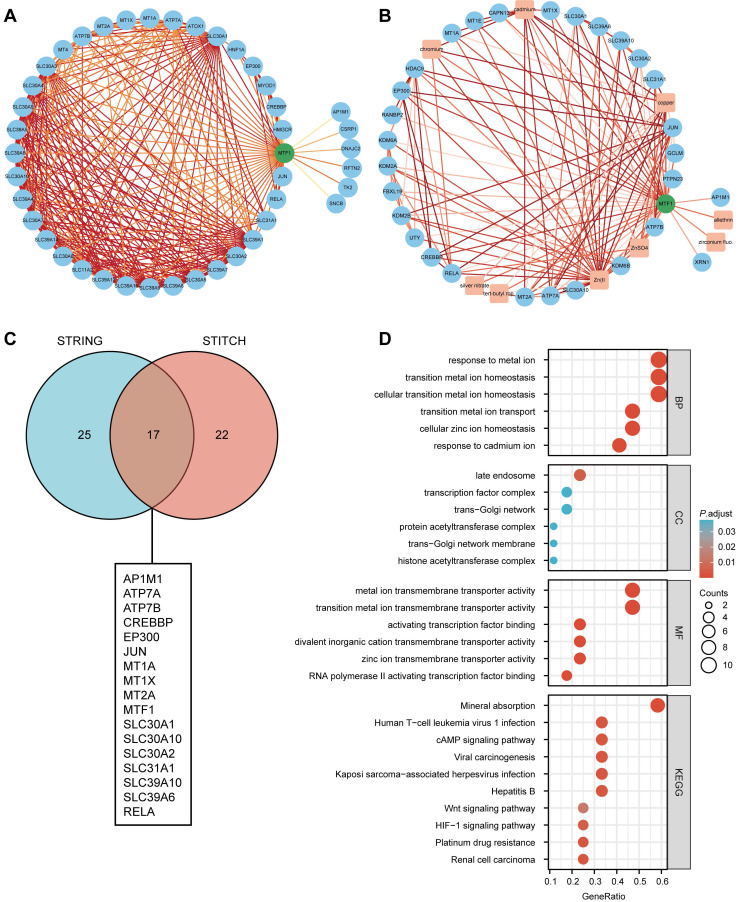
Interaction and enrichment analysis of MTF1. (**A**) PPI analysis in the STRING database. (**B**) PPI and protein-compound interaction analysis in the STITCH database. (**C**) A Venn diagram for common interactors of MTF1. (**D**) GO and KEGG pathway analysis of MTF1.

**Figure 8:**
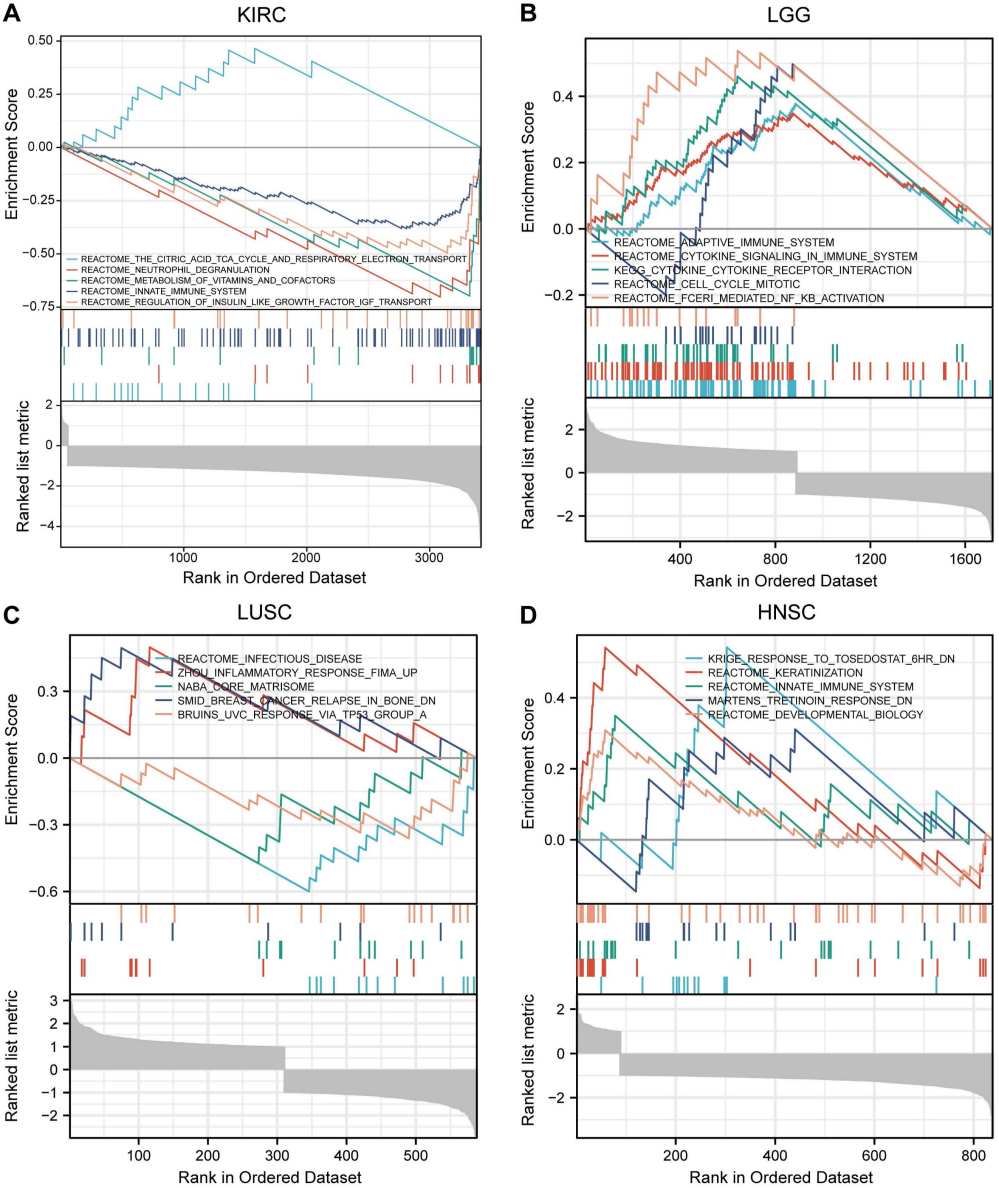
GSEA results of MTF1 in KIRC (**A**), LGG (**B**), LUSC (**C**) and HNSC (**D**).

Based on the STRING database, a PPI network of MTF1 was built. The minimum required interaction score was set to 0.4. Forty-one interactors and their interaction relationships were obtained and visualized with the help of the Cytoscape software (**Fig. 7A**). According to the STITCH database, we set the same minimum required interaction score and obtained the interaction relationship between MTF1 and 9 compounds and 29 protein molecules, which was also visualized by the Cytoscape software (**Fig. 7B**). By mapping a Venn diagram, we obtained 17 protein molecules that interact with MTF1 in both databases (**Fig. 7C**). The top enrichment results of BP, CC, MF, and KEGG are shown in a bubble chart. We found that MTF1 might play an important role in maintaining the metal ion homeostasis, constituting the late endosome and transcription factor complex, taking part in the metal ion transmembrane transport and in the mineral absorption pathway (**Fig. 7D**). To understand the probable role of MTF1 in specific cancers better, we performed the GSEA analysis in four tumors with statistical differences in survival analysis, namely KIRC, LGG, LUSC, and HNSC. The results were screened with FDR < 0.05 and adjusted *P* < 0.05 cut-offs. The screened items were then sorted by the NES from highest to lowest. We showed the top five results for each cancer species (**Fig. 8A-D**). Notably, immune system correlations were seen in the results of all of the cancers we analyzed.

### Expression Patterns and Related Functional Status of MTF1 at the Single-Cell Level

**Figure 9:**
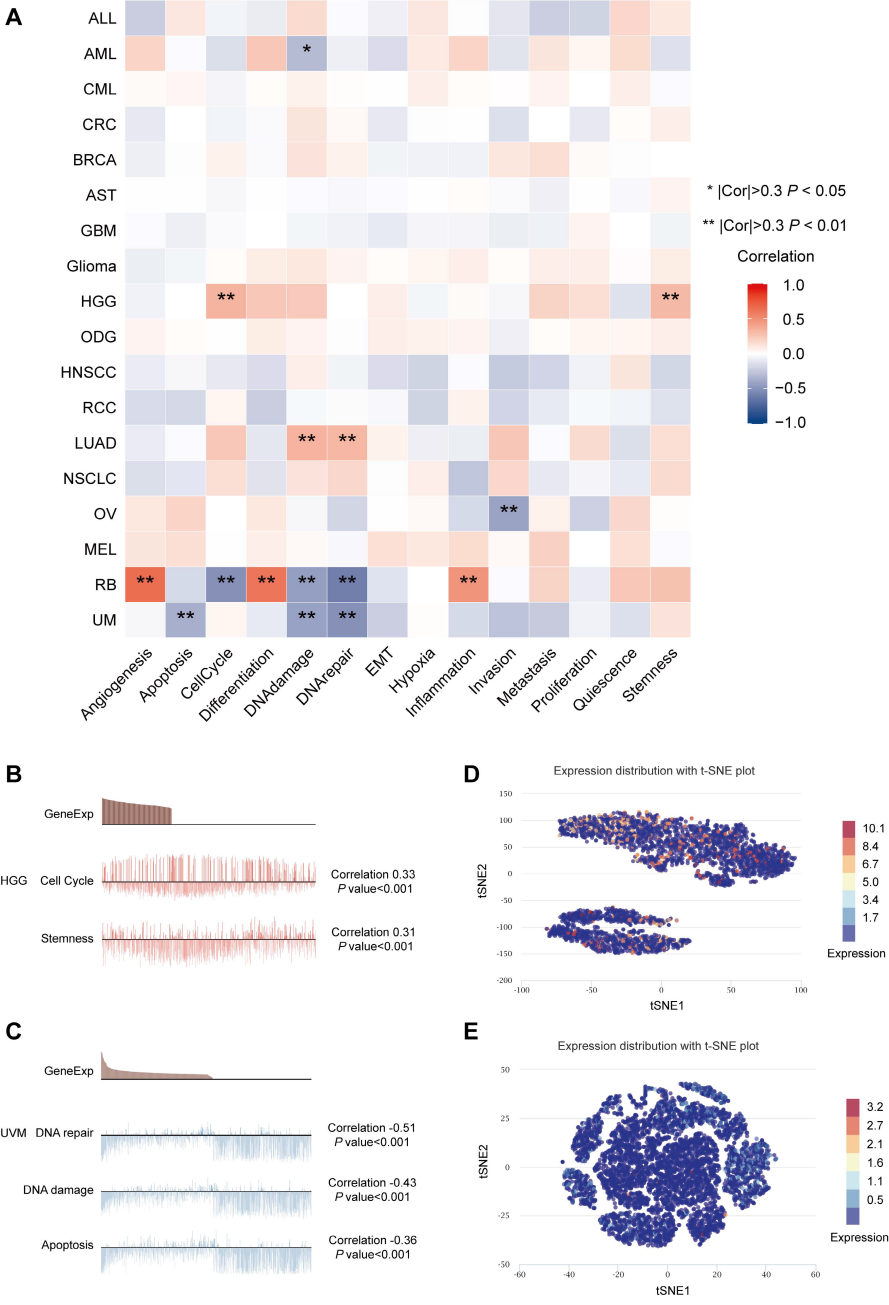
Expression patterns and related functions of MTF1 at the single-cell level. (**A**) MTF1 expression and differential functional statuses in pan-cancer. Correlation between MTF1 expression and functional statuses in HGG (**B**) and UVM (**C**). Distributions of MTF1 at the single-cell level are shown by the t-SNE diagrams in HGG (**D**) and UVM (**E**). |Cor| represents the absolute value of the correlation coefficient. * |Cor| > 0.3 and *P* < 0.05, ** |Cor| > 0.3 and *P* < 0.01.

Our results in the heatmap indicated that MTF1 had strong correlations with 14 tumors' functional status in many kinds of cancers, among which high-grade glioma (HGG), retinoblastoma (Rb), and UVM were the most significant (**Fig. 9A**). Positive relationships were shown between MTF1 expression and cell cycle and stemness in HGG (**Fig. 9B**), and negative relationships between MTF1 expression and DNA damage, DNA repair, and apoptosis were significant in UVM (**Fig. 9C**). In addition, expression distribution of MTF1 at single-cell levels is shown in t-SNE plots (**Fig. 9D, 9E**).

### Correlation Between MTF1 Expression and Immune Subtype in Pan-Cancer

**Figure 10:**
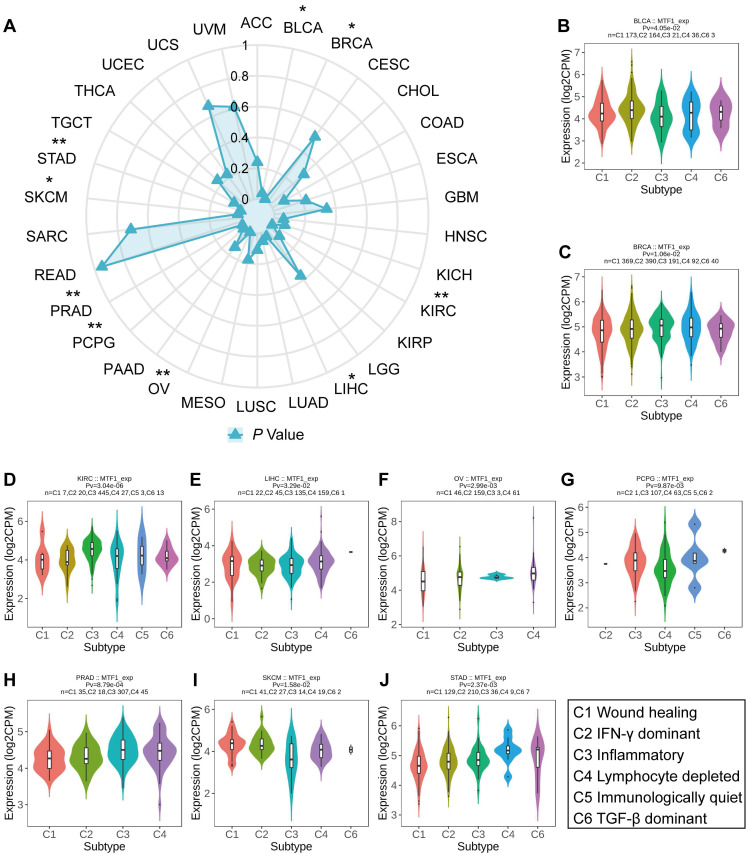
Relationship between MTF1 expression and tumor immune subtypes in pan-cancer. (**A**) A radar plot showing statistical *p*-value for the association analysis between MTF1 and immune subtypes in different tumors. MTF1 expression and tumor immune subtypes in BLCA (**B**), BRCA (**C**), KIRC (**D**), LIHC (**E**), OV (**F**), PCPG (**G**), PRAD (**H**), SKCM (**I**), and STAD (**J**). * *P* < 0.05, ** *P* < 0.01.

Based on transcriptomic profiles of more than 10,000 patients from all 33 non-hematological cancer types, a new global immunological subtype classification of solid tumors has been established and is gradually becoming widely accepted. According to this study, there are six distinct immune subtypes, namely the wound healing (C1), the IFN-g dominant (C2), the inflammatory (C3), the lymphocyte-depleted (C4), the immunologically quiet (C5), and the TGF-β dominant (C6) subtypes. We focused on whether MTF1 expression was associated with different immune subtypes of tumors and analyzed accordingly (**Fig. 10A**). The results indicated that the expression of MTF1 in BLCA, BRCA, KIRC, LIHC, OV, pheochromocytoma and paraganglioma (PCPG), READ, PRAD, SKCM, and STAD showed significant differences among different immune subtypes (*P* < 0.05) (**Fig. 10B-J**).

### Association Between MTF1 Expression and Immune Infiltration in Pan-Cancer

**Figure 11:**
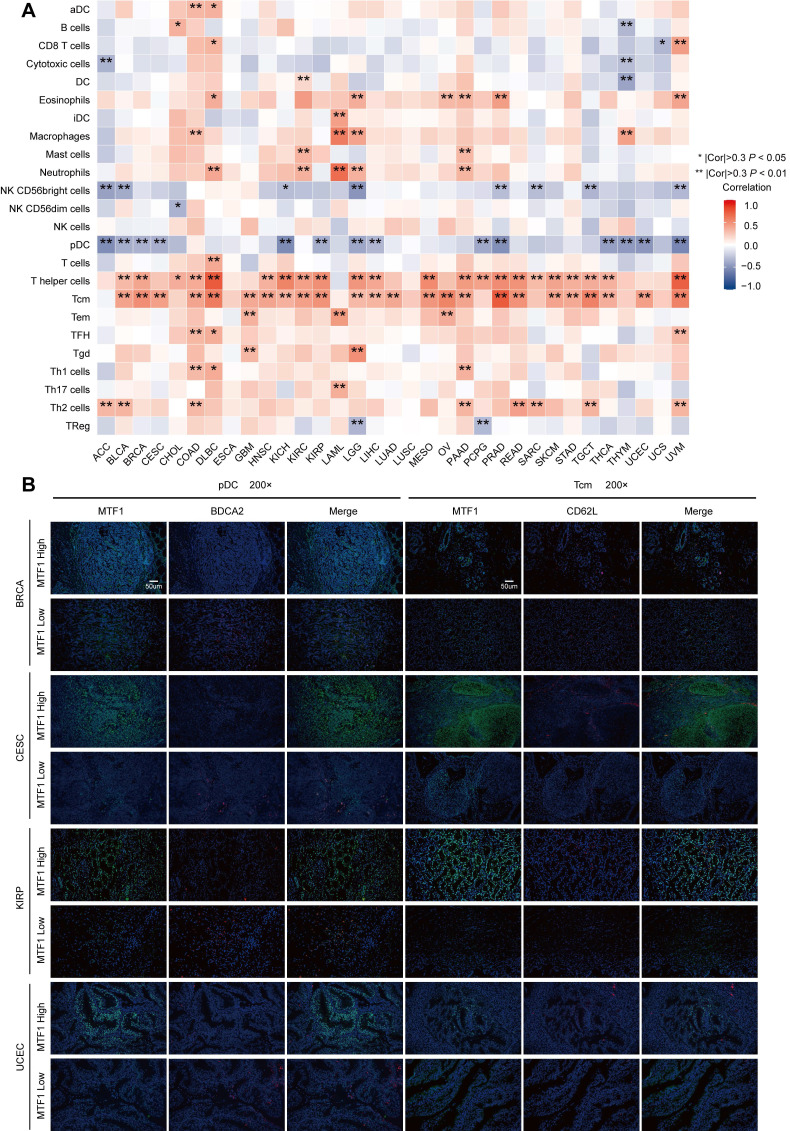
Correlation between the expression of MTF1 and immune infiltration in pan-cancer. (**A**) A heat map showing the correlation between MTF1 expression and immune cell infiltration. (**B**) The expression of MTF1 and infiltration of pDC and Tcm in BRCA, CESC, KIRP, and UCEC tissues were demonstrated by immunofluorescence at 200x magnification. |Cor| represents the absolute value of the correlation coefficient. * |Cor| > 0.3 and *P* < 0.05, ** |Cor| > 0.3 and *P* < 0.01.

The above results of enrichment analyses suggested that MTF1 was significantly correlated with innate and adaptive immunity in a variety of tumors. Therefore, we used the ssGSEA algorithm in the GSVA package (Version 1.34.0) to analyze the infiltration of 24 kinds of immune-related cells in pan-cancer. We found that the expression of MTF1 was significantly positively correlated with Tcm and T helper cells, and negatively correlated with pDC in most cancers (**Fig. 11A**). Correlation curves were shown separately when the Spearman correlation coefficient was above 0.5 (**Supplementary Fig. S6**). To verify the results of the above bioinformatics analysis, we performed immunofluorescence assays to explore the infiltration of Tcm and pDC in tumor tissue sections of BRCA, ESCA, KIRP, and UCEC. 200x microscopic images showed that MTF1 expression in tumor cells had a negative relationship to pDC infiltration and a positive relationship to Tcm infiltration, which was consistent with the above analysis results (**Fig. 11B**).

### Correlation Between MTF1 and Immune-Associated Biomarkers in Pan-Cancer

**Figure 12:**
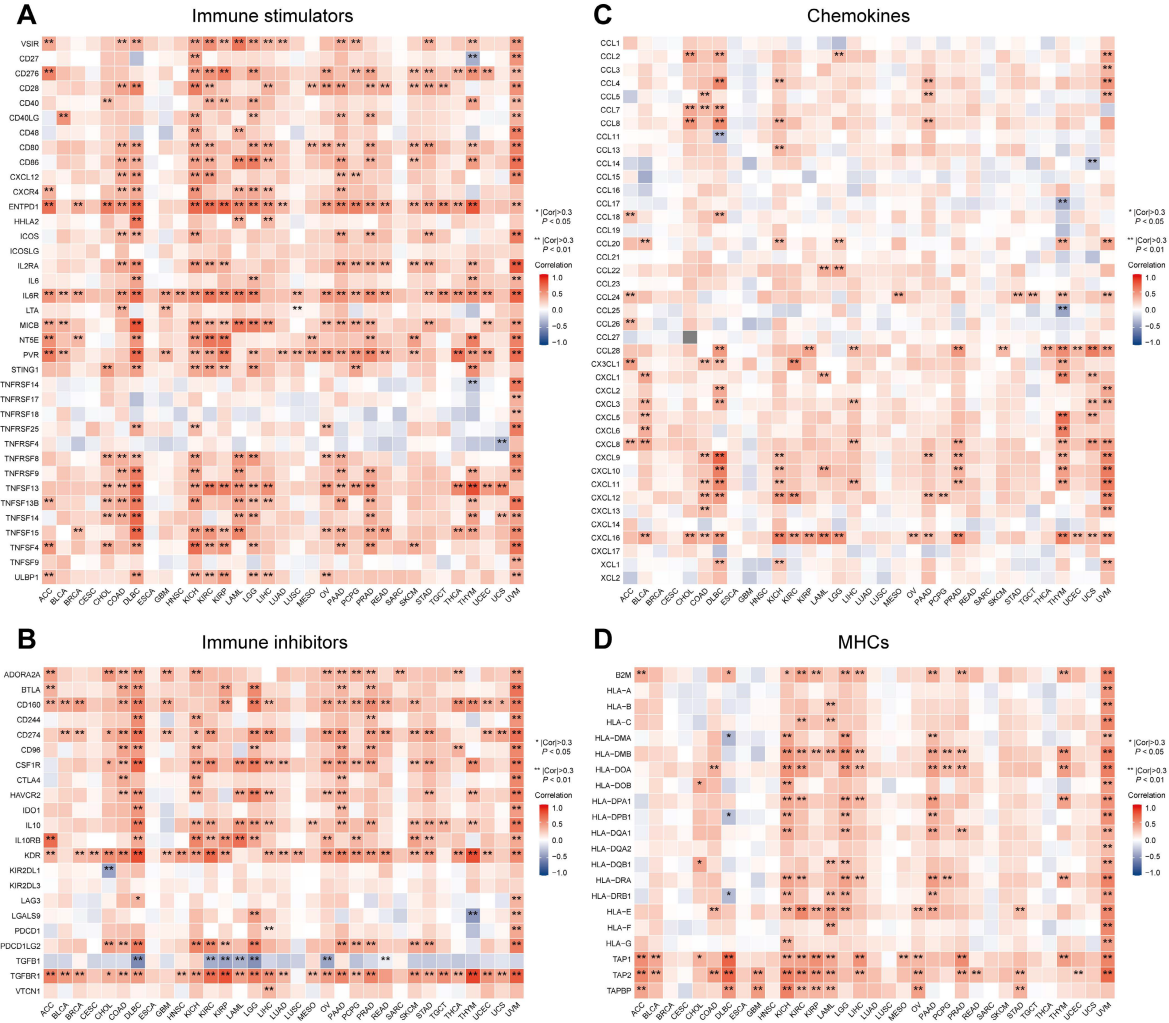
Relevance between MTF1 expression and immune-associated biomarkers in pan-cancer. Heat maps show the correlation between MTF1 expression and immune stimulators (**A**), immune inhibitors (**B**), chemokines (**C**), and MHCs (**D**). * |Cor| > 0.3 and *P* < 0.05, ** |Cor| > 0.3 and *P* < 0.01.

Besides immune cells, immune-related molecular biomarkers can also reflect the immune status of the tumor microenvironment to some extent and have the potential to predict the efficacy of immunotherapy. We applied the ggplot2 package (Version 3.3.3) to analyze and demonstrate the relationship between MTF1 expression and immune stimulators (**Fig. 12A**), immunosuppressors (**Fig. 12B**), chemokines (**Fig. 12C**), and MHCs (**Fig. 12D**) in different cancers. Spearman correlation greater than 0.3 and *P*-values less than 0.05 were considered statistically significant and marked with “*” or “**” on the heatmaps.

### MTF1 Inhibits Cuproptosis In Vitro

**Figure 13:**
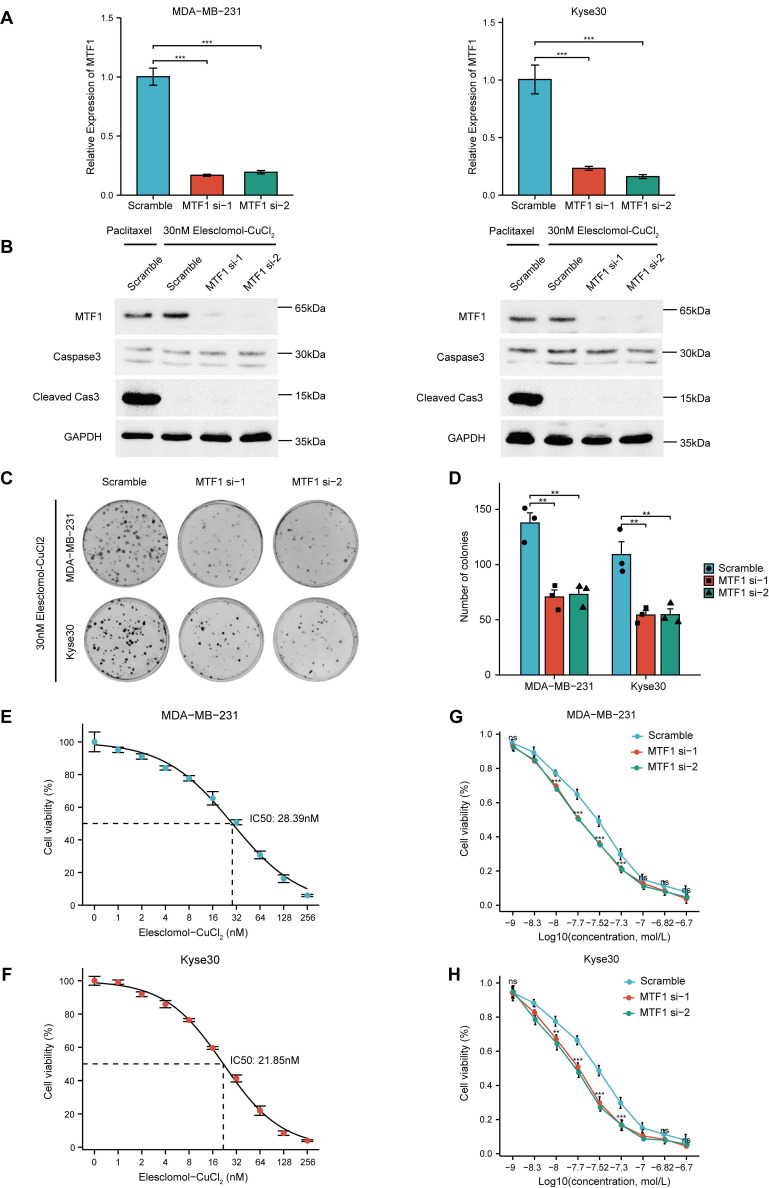
MTF1 reduced sensitivity of tumor cells to cuproptosis in vitro. (**A**) Validation of interference efficiency of siRNA for MTF1 expression via q-PCR. (**B**) Expression of apoptosis markers in different cells after different treatments. (**C**) Colony formation images of MDA-MB-231 and Kyse30 under Elesclomol-CuCl2 (1:1) treatment. (**D**) Statistical graph of colony formation assay. IC50 estimation of MDA-MB-231 (**E**) and Kyse30 (**F**) to Elesclomol-CuCl2 (1:1). Cell viability curves under treatment of different concentrations of Elesclomol-CuCl2 (1:1) in MDA-MB-231 (**G**) and Kyse30 (**H**). * *P* < 0.05, ** *P* < 0.01, *** *P* < 0.001.

Given that the reported association between MTF1 and cuproptosis was based only on high-throughput screening and bioinformatics analysis, we conducted relevant cell assays to explore the relationship. Firstly, we used siRNAs to knock down MTF1 in two moderately expressed cell lines (MDA-MB-231 and Kyse30) and verified their efficiency by qRT-PCR experiments (**Fig. 13A**). Subsequently, 30nM Elesclomol-CuCl_2_ (1: 1) was used to treat cells in the exponential growth phase, and apoptosis-related biomarkers caspase3 and cleaved caspase3 were detected by western blotting. The appropriate concentration of paclitaxel was set as the positive control in the above experimental groups. The results showed that cuproptosis-inducing treatment could not induce apoptosis of cells (**Fig. 13B**). Colony formation assay showed that MTF1 knockdown significantly impaired the clonogenic ability of cells treated with 30nM Elesclomol-CuCl_2_ (**Fig. 13C, 13D**) (*P* < 0.01). The median lethal dose (IC50) of Elesclomol-CuCl_2_ in two kinds of cells was determined by CCK8 assay. The IC50 of MDA-MB-231 was 28.39nM (**Fig. 13E**) and the IC50 of Kyse30 was 21.85nM (**Fig. 13F**). Furthermore, the survival of MTF1-knockdown cells and control cells at different concentrations of Elesclomol-CuCl_2_ (1nM, 5nM, 10nM, 20nM, 30nM, 50nM, 100nM, 150nM, and 200nM) were detected and their respective curves were drawn. We found that at certain drug concentrations such as 10nM, 20nM, 30nM, and 50nM, the cell viability rate of MTF1-knockdown cells was significantly lower than that of control cells (*P* < 0.05) (**Fig. 13G, 13H**). This suggested that MTF1 could reduce the sensitivity of cells to cuproptosis induction conditions.

## Discussion

Metal ions have been proven to be indispensable structural components of many proteins or intrinsic cofactors of various enzymes and play a wide range of biological roles in the body 31, 32. Therefore, metal ion homeostasis in the body needs precise and timely regulation, and as one of the most important effector elements in this process, the role of MTF1 in different diseases deserves further study.

In recent years, malignant tumors and immunotherapy have been two topics that a great number of researchers are keen to explore. Immunotherapy has shown great potential and advantages in patients with a variety of refractory malignant tumors who have poor responses to conventional treatments such as chemotherapy and radiotherapy. It is worth noting that metal ions are extremely closely related to both. Therefore, we hope to have a deep and comprehensive understanding of the role of MTF1 in cancer and immunity.

High expression of MTF-1 was proven to increase zinc-induced activation of ERK1/2 and AKT, thereby inducing the progression of ovarian cancer 33. However, reports on MTF1 in most kinds of cancers are scarce so far. In this study, by analyzing MTF1 mRNA levels in paired and unpaired samples, we found that MTF1 expression in most tumors was different from that in normal tissues and closely related to cancer severity and prognosis, pathological stage, histological grade, and the outcome of initial treatment. In addition, the ROC curve indicated the diagnostic role of MTF1 in pan-cancer. Survival is one of the most important indicators of all malignancies. In this study, we found the prognostic value of MTF1 in LGG, HNSC, KIRC, READ, ACC, LUSC, and SKCM. Univariate and multivariate Cox regression analyses identified MTF1 as an independent prognostic factor for KIRC and LGG.

Methylation and CNV are two of the most important factors affecting gene expression 34. We found that methylation and CNV of MTF1 were different in various tumors and normal cells, and were significantly related to its expression and survival, which may provide clues to explain the abnormal expression of MTF1 in various tumors and warrant further study. Mutations, which are widely present in many diseases including cancer, can affect the conformation and function of proteins and cause a series of pathological processes without abnormal expression 35. Here we uncovered that MTF1 had a considerable mutation rate in a variety of malignant tumors, and some of them were correlated with prognosis and other indicators. As the roles of wild-type and mutant MTF1 in different physiological and pathological processes are further revealed, corresponding drugs can be designed accordingly.

Previous studies have reported the role of MTF1 in several physiological and pathological processes; for example, it influences oxidative stress and inflammation of alcoholic hepatitis hepatocytes by regulating the expression of metallothionein 1 and 2 36, and it promotes myogenesis in response to copper 37. In this study, a series of enrichment analyses were conducted to explore the functions of MTF1 in cancer. Innate immunity, adaptive immunity, cell cycle, inflammatory response, and keratinization were enriched in GSEA results of various cancers, which was consistent with the GO and KEGG pathway enrichment analysis and previous research.

The great regulatory effect of metal ions on the immune system has been widely studied and accepted by researchers. However, as an important molecule involved in the regulation of intracellular metal ion homeostasis, MTF1 has been limitedly reported to be related to human immunity previously. We first analyzed the association between MTF1 expression and cancer immune subtypes, which was reported by Akinyemi I et al. for the first time, and has been widely used in cancer immunotyping. According to our results, MTF1 expression was discrepant in different immune subtypes of nine cancers, and in tumors including BLCA, BRCA, KIRC, PRAD, and SKCM, MTF1 expression was higher in C2 (IFN-γ dominant) or C3 (inflammatory) and lower in C6 (TGF-β) subtype, which was consistent with previous findings 38.

Further analyses targeted more specific immune cells and immune-related molecules which might play a key role in antitumor immunity 39, 40. Through a widely used ssGSEA method, we found that MTF1 was significantly negatively correlated with pDC and positively correlated with helper T cell and Tcm in most cancers. pDCs developed from both common DC precursors and lymphoid progenitors can express CD123, CD303 (BDCA2), CD304, and CXCR3 as superficial markers and may have an antiviral effect 41, 42. However, the activity of pDC has been reported to be inhibited in most tumor microenvironments and associated with poor prognosis in a variety of tumors, which may result from the ability of pDCs to promote the expansion of Treg cells in an inducible T cell co-stimulator ligand (ICOSL)-dependent manner 43. Tcm are long-term memory T cells expressing CD62L and CD40RO 44. Recent studies have shown that Tcm plays an antitumor role in the early stage of immunotherapy and can improve the efficacy of immunotherapy for some cancers 45, 46. We verified the negative relationship between MTF1 and pDC and the positive relationship between MTF1 and Tcm in four tumors. Along with the correlations between MTF1 expression and immune-associated molecules, including immune stimulators, immune inhibitors, chemokines, and MHCs, we suggested that MTF1 may be predictive of the tumor immune microenvironment and immunotherapy efficacy in a variety of cancers.

Regulatory cell death, such as ferroptosis and pyroptosis, has been widely studied in recent years and reported as potential ways to improve cancer treatment 47-49. Similar to ferroptosis, there is also a large amount of reactive oxygen species (ROS) produced during cuproptosis 10. In the last part of this study, we performed a series of experiments and demonstrated that the reduction of MTF1 could increase the sensitivity of tumor cells to cuproptosis. As the role of cuproptosis in cancer treatment is gradually revealed 50, the cuproptosis-suppressive molecule MTF1 may be a potential therapeutic target for some cancers.

Nevertheless, there are still some limitations in this study. To begin with, most of the results obtained in this research were based on bioinformatics analysis methods, and further in vivo and in vitro experiments are needed to prove their reliability. Second, this study failed to give a more specific explanation for the different expression and survival of MTF1 in pan-cancer. Third, this study applied a lot of correlation analyses and obtained corresponding results, but we cannot determine whether MTF1 and those related variables have direct or indirect regulatory relations. Fourth, in cell experiments, the selection of cell lines is not representative enough, and the grouping lacks overexpression groups. Given that MTF1 is dysregulated in a variety of cancers and its probable role in immune and cell death, many problems remain to be investigated in the future.

## Conclusions

In conclusion, MTF1 is likely to be a potential biomarker for diagnosis and prognosis of a variety of cancers. Meanwhile, the correlations between MTF1 and immune cell infiltration and the expression of immune-related molecules in the tumor microenvironment suggested that MTF1 might play a role in immune regulation and immunotherapy response prediction in some cancers. The inhibitory effect of MTF1 on cuproptosis, one of the novel regulatory cell death modes in tumor cells, indicated that MTF1 may be a potential therapeutic target for some refractory cancers in the future.

## Supplementary Material

Supplementary figures and tables.

## Figures and Tables

**Table 1:** Univariate and multivariate Cox regression analysis of KIRC

Clinical characteristics	Total (N)	Univariate analysis		Multivariate analysis	
		HR (95% CI)	*P*-value	HR (95% CI)	*P*-value
Age (>60 vs. <=60) ^a^	539	1.765 (1.298-2.398)	**<0.001**	1.647 (1.075-2.522)	**0.022**
Gender (Male vs. Female)	539	0.930 (0.682-1.268)	0.648		
T stage (T3&T4 vs. T1&T2) ^b^	539	3.228 (2.382-4.374)	**<0.001**	1.905 (0.819-4.432)	0.135
N stage (N1 vs. N0) ^b^	257	3.453 (1.832-6.508)	**<0.001**	1.582 (0.780-3.208)	0.204
M stage (M1 vs. M0)^ b^	506	4.389 (3.212-5.999)	**<0.001**	3.106 (1.791-5.386)	**<0.001**
Pathologic stage (III&IV vs. I&II)	536	3.946 (2.872-5.423)	**<0.001**	1.006 (0.385-2.627)	0.990
Histologic grade (G3&G4 vs. G1&G2)	531	0.680 (0.562-0.823)	**<0.001**	1.517 (0.913-2.520)	0.108
MTF1	539	0.680 (0.562-0.823)	**<0.001**	0.667 (0.493-0.902)	**0.009**

^a^ Cut-off value based on previous studies. ^b^ Diagnosed based on the AJCC, 2017 criteria (the eighth edition).

**Table 2:** Univariate and multivariate Cox regression analysis of LGG

Clinical characteristics	Total (N)	Univariate analysis		Multivariate analysis	
		HR (95% CI)	*P*-value	HR (95% CI)	*P*-value
Age (>40 vs. <=40) ^a^	509	2.868 (1.962-4.194)	**<0.001**	2.814 (1.791-4.420)	**<0.001**
Gender (Male vs. Female)	509	1.094 (0.767-1.561)	0.619		
WHO grade (G3 vs. G2) ^b^	452	3.167 (2.071-4.842)	**<0.001**	2.095 (1.325-3.314)	**0.002**
Histological type ^c^	320	0.614 (0.384-0.982)	**0.042**	1.321 (0.789-2.211)	0.290
Histological type ^d^	381	0.558 (0.374-0.834)	**0.004**	0.898 (0.558-1.446)	0.659
IDH status (Mut vs. WT) ^e^	506	0.155 (0.107-0.225)	**<0.001**	0.237 (0.150-0.376)	**<0.001**
MTF1	509	2.640 (1.796-3.881)	**<0.001**	1.801 (1.191-2.722)	**0.005**

^a^ Cut-off value based on previous studies. ^b^ According to the 2021 WHO classification. ^c^ Oligoastrocytoma vs. astrocytoma ^d^ Oligodendroglioma vs. astrocytoma ^e^ Mut: mutation, WT: wild type

## Abbreviations

MTF1: Metal regulatory transcription factor 1
pDC: plasmacytoid dendritic cells
Tcm: central memory T cells
MRE: metal response elements
TCA: tricarboxylic acid
GTEx: the Genotype-Tissue Expression
TCGA: The Cancer Genome Atlas
UCSC Xena: the University of California Santa Cruz
FPKM: fragments per kilobase per million
TPM: transcripts per million reads
ROC: receiver operating characteristic
AUC: area under curve
OS: overall survival
PFS: progression-free survival
DSS: disease-specific survival
KIRC: kidney renal clear cell carcinoma
LGG: brain lower grade glioma
CNV: copy number variation
GSCA: gene set cancer analysis
HNSC: head and neck squamous cell carcinoma
LUSC: lung squamous cell carcinoma
GDSC: the Genomics of Drug Sensitivity in Cancer
CTRP: the Cancer Therapeutics Response Portal
GAPDH: glyceraldehyde-3-phosphate dehydrogenase
STRING: The Search Tool for the Retrieval of Interacting Genes/Proteins
PPI: Protein-Protein Interaction
STITCH: the search tool for interacting chemicals
GO: Gene Ontology
KEGG: Kyoto Encyclopedia of Genes and Genomes
BP: biological process
CC: cellular component
MF: molecular function
GSEA: Gene Set Enrichment Analysis
FDR: false discovery rate
NES: normalized enrichment score
EMT: epithelial-mesenchymal transition
t-SNE: the t-distributed stochastic neighbor embedding
IFN-γ: interferon-γ
TGF-β: transforming growth factor-β
aDC: activated dendritic cells
DC: dendritic cells
iDC: immature dendritic cells
NK: natural killer cells
p-DC: plasmacytoid dendritic cells
Tcm: central memory T cells
Tem: effector memory T cells
Tfh: follicular helper T cells
Treg: regulatory T cells
ssGSEA: single sample gene set enrichment analysis
MHCs: major histocompatibility complexes
TSA: tyrosine signal amplification
ACC: adrenocortical carcinoma
BLCA: bladder urothelial carcinoma
BRCA: breast invasive carcinoma
CHOL: cholangiocarcinoma
DLBC: diffuse large B-cell lymphoma
ESCA: esophageal carcinoma
GBM: glioblastoma multiforme
KICH: kidney chromophobe
KIRP: kidney renal papillary cell carcinoma
LAML: acute myeloid leukemia
LIHC: liver hepatocellular carcinoma
LUAD: lung adenocarcinoma
OV: ovarian serous cystadenocarcinoma
PAAD: pancreatic adenocarcinoma
SKCM: skin cutaneous melanoma
STAD: stomach adenocarcinoma
TGCT: testicular germ cell tumors
THCA: thyroid carcinoma
THYM: thymoma
UCEC: uterine corpus endometrial carcinoma
UCS: uterine carcinosarcoma
COAD: colon adenocarcinoma
CR: complete response
PRAD: prostate adenocarcinoma
HR: hazard ratio
CESC: endocervical adenocarcinoma
MESO: mesothelioma
SARC: sarcoma
READ: rectum adenocarcinoma
UVM: uveal melanoma
HGG: high-grade glioma
Rb: retinoblastoma
PCPG: pheochromocytoma and paraganglioma
qRT-PCR: Quantitative Real-Time PCR
IC50: median lethal dose
