# Supplementary material for: Prognostic and Immunological Role of Cuproptosis-Related Gene MTF1 in Pan-Cancer
Source: J Cancer. 2024 Sep 9;15(17):5786–809. doi: 10.7150/jca.98749 (PMC11414622; doi:10.7150/jca.98749)
Supplement: Supplementary file 1 — Supplementary figures and tables. [file jcav15p5786s1.pdf]

Supplementary Material

Supplementary Tables

Supplementary TableS1: Baseline data for KIRC patients

Characteristics	Expression of MTF1		*P* value
	Low	High	
Age[a], N=539	269	270	0.282
<= 60	128 (23.7%)	140 (26.0%)	
> 60	141 (26.2%)	130 (24.1%)	
Gender, N=539	269	270	**0.006**
Male	191 (35.4%)	161 (29.9%)	
Female	78 (14.5%)	109 (20.2%)	
Pathologic T stage[b], N=539	269	270	**0.001**
T1&T2	156 (28.9%)	192 (35.6%)	
T3&T4	113 (21.0%)	78 (14.5%)	
Pathologic N stage[b], N=257	128	129	0.97
N0	120 (46.7%)	121 (47.1%)	
N1	8 (3.1%)	8 (3.1%)	
Pathologic M stage[b], N=506	253	253	0.111
M0	207 (40.9%)	220 (43.5%)	
M1	46 (9.1%)	33 (6.5%)	
Pathologic stage, N=536	268	268	**<0.001**
Stage I & Stage II	144 (26.9%)	186 (34.7%)	
Stage III & Stage IV	124 (23.1%)	82 (15.3%)	
Histologic grade, N=531	265	266	**<0.001**
G1&G2	102 (19.2%)	146 (27.5%)	
G3&G4	163 (30.7%)	120 (22.6%)	

[a] Cut-off value based on previous studies.

[b] Diagnosed based on the AJCC, 2017 criteria (the eighth edition).

Supplementary TableS2: Baseline data for LGG patients

**Characteristics**	**Expression of MTF1**		***P* value**
	**Low**	**High**	
Age[a], N=509	254	255	**0.030**
<= 40	112 (22.0%)	137 (26.9%)	
> 40	142 (27.9%)	118 (23.2%)	
Gender, N=509	254	255	0.199
Female	120 (23.6%)	106 (20.8%)	
Male	134 (26.3%)	149 (29.3%)	
WHO grade[b], N=452	226	226	**<0.001**
G2	128 (28.3%)	89 (19.7%)	
G3	98 (21.7%)	137 (30.3%)	
Histological type, N=509	254	255	**<0.001**
Oligoastrocytoma	54 (10.6%)	74 (14.5%)	
Astrocytoma	61 (12.0%)	131 (25.7%)	
Oligodendroglioma	139 (27.3%)	50 (9.8%)	
IDH status[c], N=506	253	253	**<0.001**
WT	28 (5.5%)	66 (13.0%)	
Mut	225 (44.5%)	187 (37.0%)	

[a] Cut-off value based on previous studies.

[b] According to the 2021 WHO classification.

[c] Mut: mutation, WT: wild type

Supplementary Figures:

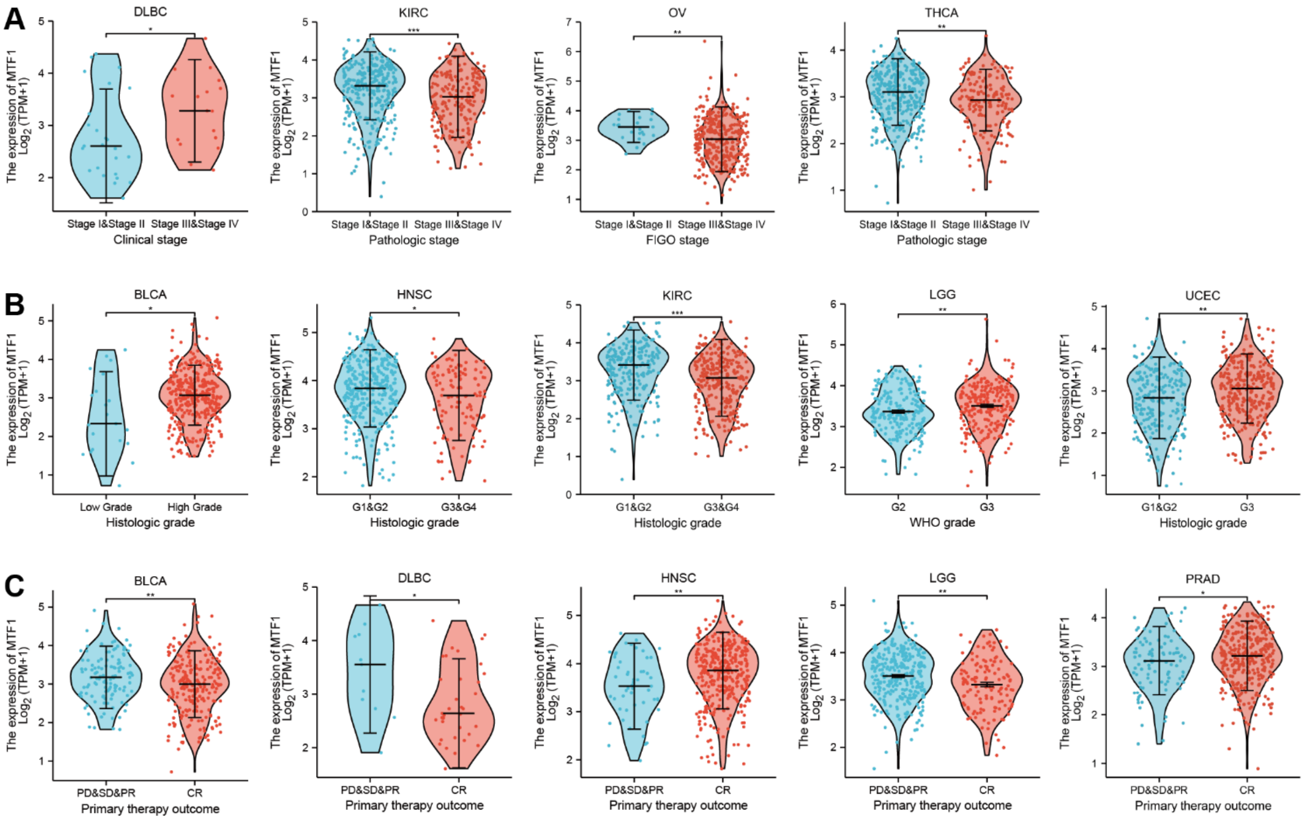

Supplementary Fig. S1. Expression of MTF1 was correlated with clinicopathological features in various cancers. (**A**) MTF1 expression was related to the stage in DLBC, KIRC, OV and THCA. (**B**) MTF1 expression was related to grade in BLCA, HNSC, KIRC, LGG and UCEC. (**C**) MTF1 expression was related to the primary therapy outcome in BLCA, DLBC, HNSC, LGG and PRAD. * $P < 0.05$, ** $P < 0.01$, *** $P < 0.001$.

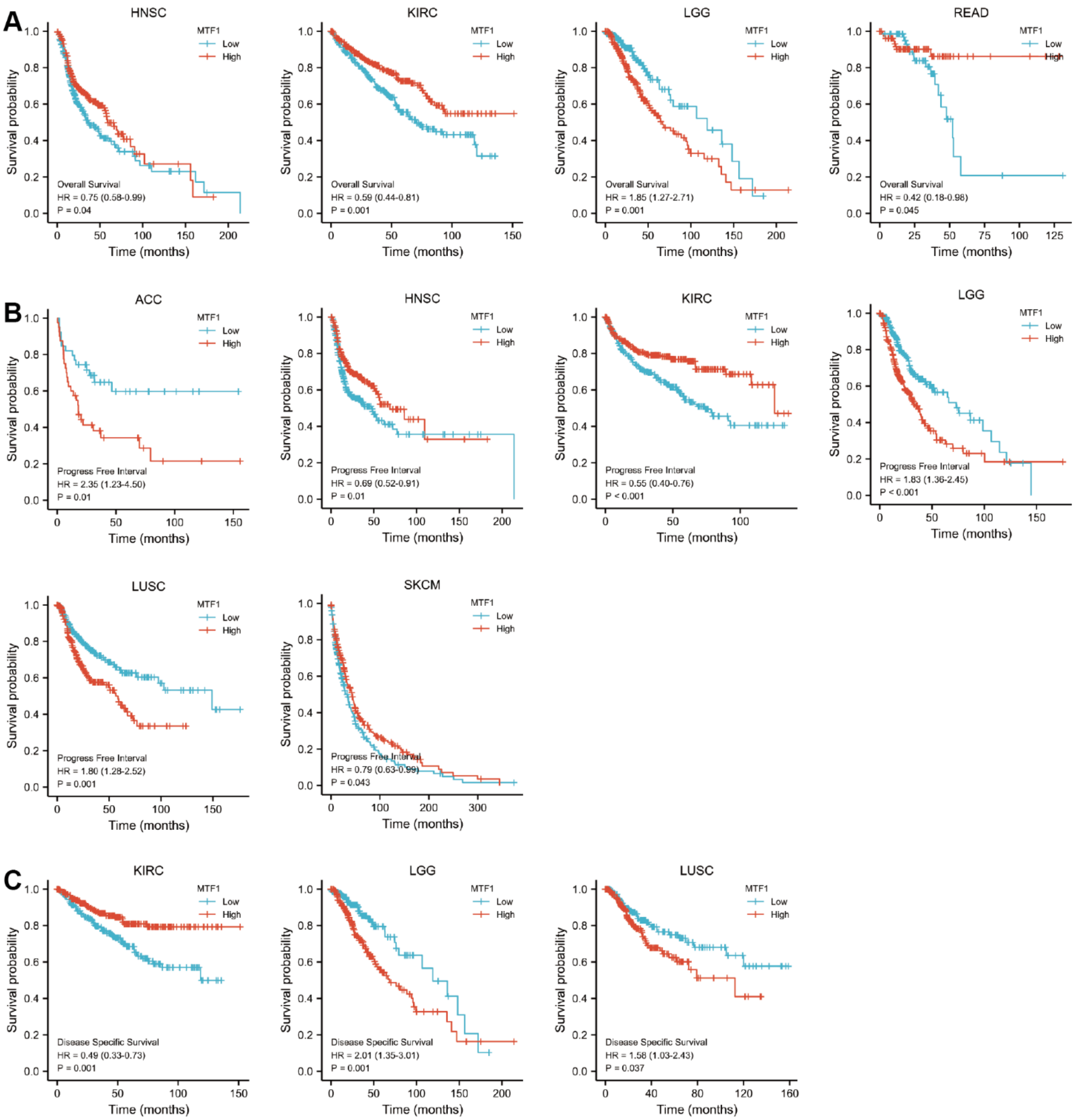

Supplementary Fig. S2. Expression of MTF1 was correlated with survival across cancers. (**A**) K-M plots of OS analysis in HNSC, KIRC, LGG and READ. (**B**) K-M plots of PFS analysis in ACC, HNSC, KIRC, LGG, LUSC and SKCM. (**C**) K-M plots of DSS analysis in KIRC, LGG and LUSC.

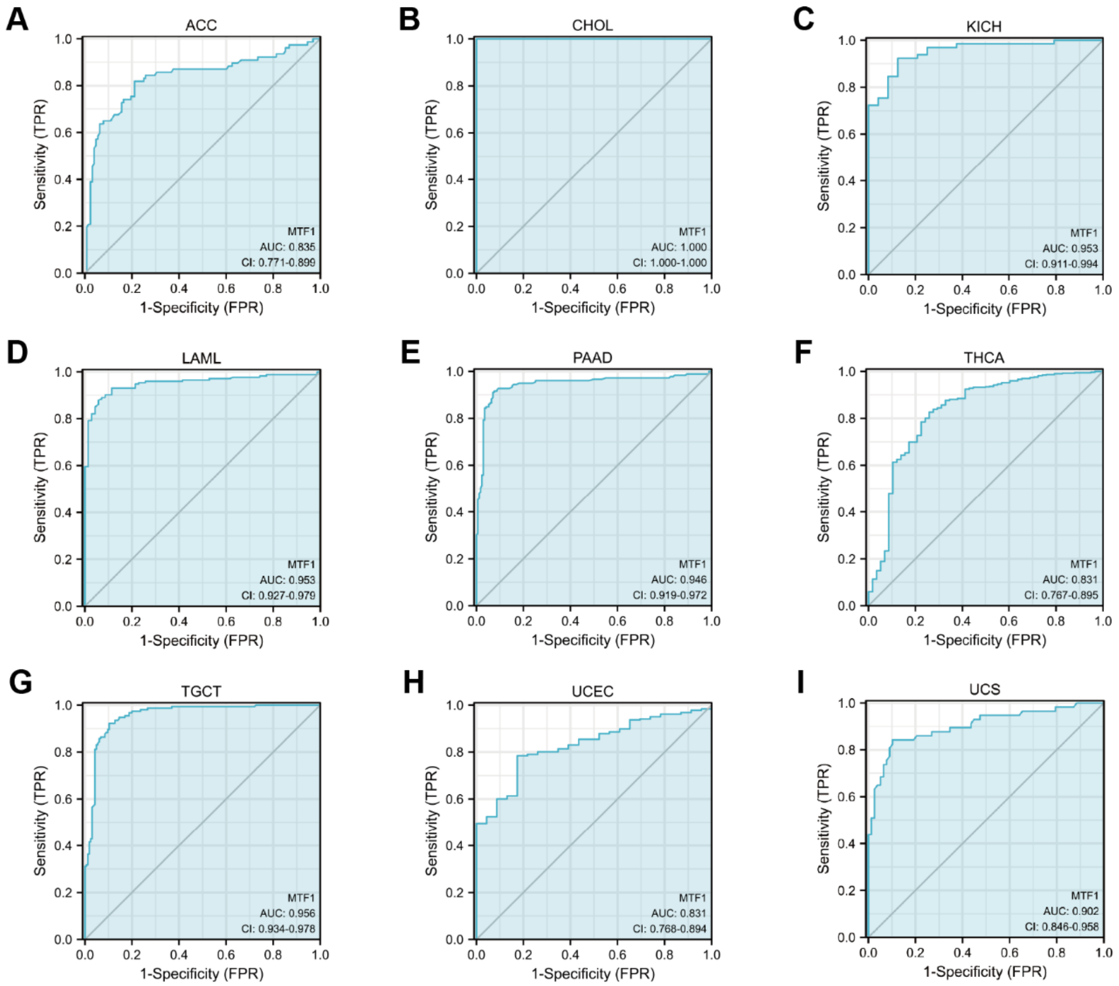

Supplementary Fig. S3. MTF1 expression had diagnostic value in a variety of cancers. ROC curves in ACC (**A**), CHOL (**B**), KICH (**C**), LAML (**D**), PAAD (**E**), THCA (**F**), TGCT (**G**), UCEC (**H**), UCS (**I**).

Cancer	Name	Relation to CpG Island	HR (CI 95%)	*P* value
KIRC	cg00977164	N_Shore	0.585 (0.384;0.892)	0.013
KIRC	cg04304494	Island	0.625 (0.424;0.921)	0.018
KIRC	cg04496824	S_Shelf	1.18 (0.802;1.736)	0.401
KIRC	cg07031996	N_Shore	1.366 (0.859;2.171)	0.187
KIRC	cg07931069	Island	0.661 (0.438;0.999)	0.049
KIRC	cg11584499	N_Shelf	2.82 (1.539;5.165)	0.001
KIRC	cg12477165	N_Shore	0.366 (0.204;0.656)	0.001
KIRC	cg13470367	S_Shore	0.657 (0.403;1.069)	0.091
KIRC	cg13567986	Open_Sea	2.45 (1.394;4.305)	0.002
KIRC	cg13987712	Island	1.211 (0.762;1.925)	0.418
KIRC	cg16249798	Island	0.462 (0.27;0.788)	0.005
KIRC	cg16707128	Island	1.676 (1.123;2.503)	0.012
KIRC	cg19895474	Island	0.565 (0.349;0.916)	0.021
KIRC	cg27628961	Open_Sea	0.65 (0.441;0.959)	0.03
LGG	cg00977164	N_Shore	1.242 (0.81;1.903)	0.321
LGG	cg04304494	Island	1.201 (0.838;1.723)	0.319
LGG	cg04496824	S_Shelf	0.427 (0.3;0.608)	<0.001
LGG	cg07031996	N_Shore	2.805 (1.915;4.108)	<0.001
LGG	cg07931069	Island	1.384 (0.973;1.97)	0.071
LGG	cg11584499	N_Shelf	0.794 (0.539;1.169)	0.243
LGG	cg12477165	N_Shore	0.683 (0.475;0.982)	0.04
LGG	cg13470367	S_Shore	0.47 (0.288;0.766)	0.002
LGG	cg13567986	Open_Sea	0.481 (0.301;0.769)	0.002
LGG	cg13987712	Island	1.296 (0.886;1.895)	0.181
LGG	cg16249798	Island	0.753 (0.522;1.088)	0.131
LGG	cg16707128	Island	0.5 (0.336;0.746)	0.001
LGG	cg19895474	Island	1.405 (0.942;2.096)	0.095
LGG	cg27628961	Open_Sea	1.744 (1.204;2.526)	0.003
HNSC	cg00977164	N_Shore	0.843 (0.618;1.15)	0.28
HNSC	cg04304494	Island	0.88 (0.644;1.201)	0.42
HNSC	cg04496824	S_Shelf	0.803 (0.596;1.081)	0.148
HNSC	cg07031996	N_Shore	0.741 (0.553;0.993)	0.045
HNSC	cg07931069	Island	0.833 (0.609;1.139)	0.251
HNSC	cg11584499	N_Shelf	1.372 (1.029;1.831)	0.031
HNSC	cg12477165	N_Shore	1.135 (0.837;1.539)	0.414
HNSC	cg13470367	S_Shore	1.223 (0.922;1.623)	0.162
HNSC	cg13567986	Open_Sea	0.733 (0.562;0.955)	0.021
HNSC	cg13987712	Island	0.84 (0.623;1.132)	0.251
HNSC	cg16249798	Island	0.811 (0.603;1.09)	0.164
HNSC	cg16707128	Island	1.116 (0.857;1.454)	0.415
HNSC	cg19895474	Island	1.218 (0.876;1.693)	0.241
HNSC	cg27628961	Open_Sea	1.125 (0.854;1.483)	0.403
LUSC	cg00977164	N_Shore	1.328 (0.913;1.932)	0.138
LUSC	cg04304494	Island	1.237 (0.854;1.792)	0.261
LUSC	cg04496824	S_Shelf	1.091 (0.763;1.561)	0.632
LUSC	cg07031996	N_Shore	0.758 (0.552;1.042)	0.088
LUSC	cg07931069	Island	1.316 (0.909;1.903)	0.145
LUSC	cg11584499	N_Shelf	1.23 (0.887;1.705)	0.215
LUSC	cg12477165	N_Shore	0.726 (0.519;1.015)	0.061
LUSC	cg13470367	S_Shore	1.072 (0.749;1.532)	0.704
LUSC	cg13567986	Open_Sea	1.202 (0.871;1.66)	0.263
LUSC	cg13987712	Island	0.885 (0.632;1.24)	0.478
LUSC	cg16249798	Island	0.88 (0.62;1.25)	0.476
LUSC	cg16707128	Island	0.804 (0.568;1.139)	0.219
LUSC	cg19895474	Island	1.122 (0.814;1.548)	0.481
LUSC	cg27628961	Open_Sea	1.44 (1.019;2.036)	0.039

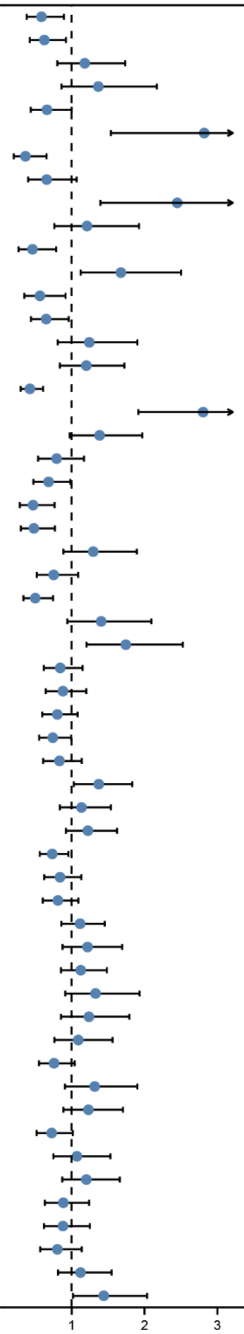

Supplementary Fig. S4. A forest map of risk assessment for different methylation sites in KIRC, LGG, HNSC and LUSC.

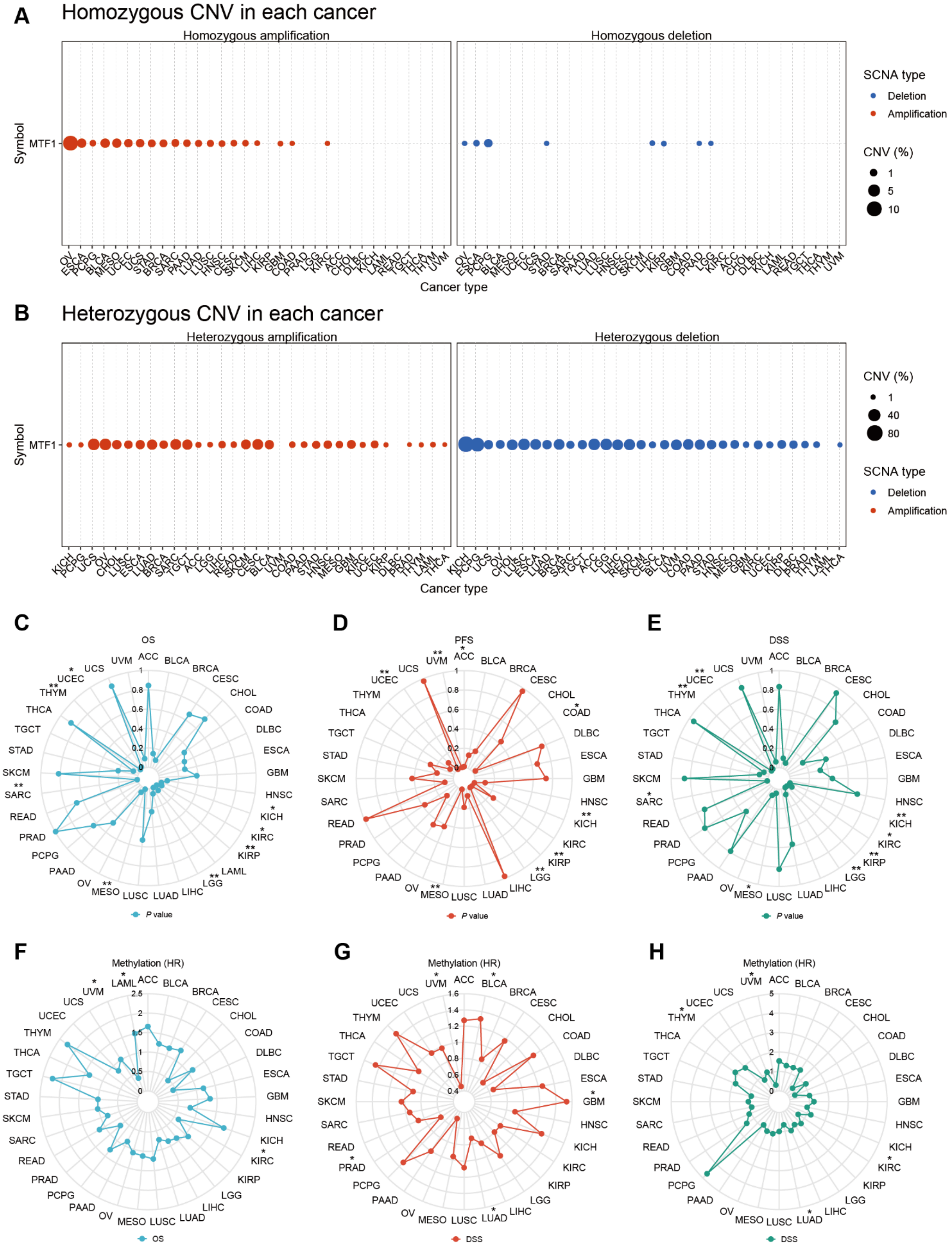
A
Homozygous CNV in each cancer
Homozygous amplification
Homozygous deletion
Symbol
MTF1
Cancer type
SCNA type
Deletion
Amplification
CNV (%)
1
5
10
B
Heterozygous CNV in each cancer
Heterozygous amplification
Heterozygous deletion
Symbol
MTF1
Cancer type
CNV (%)
1
40
80
SCNA type
Deletion
Amplification
C
OS
P value
D
PFS
P value
E
DSS
P value
F
Methylation (HR)
OS
G
Methylation (HR)
DSS
H
Methylation (HR)
DSS

Supplementary Figure S5. CNV and methylation associated analyses of MTF1 gene. (**A**) Homozygous CNV profiles in pan-cancer. (**B**) Heterozygous CNV profiles in pan-cancer. P value of correlation analyses between survival including the OS (**C**), PFS (**D**) and DSS (**E**) and CNV. Correlation coefficient analyses between survival including the OS (**F**), PFS (**G**) and DSS (**H**) and methylation. * $P < 0.05$, ** $P < 0.01$.

Supplementary Figure S6

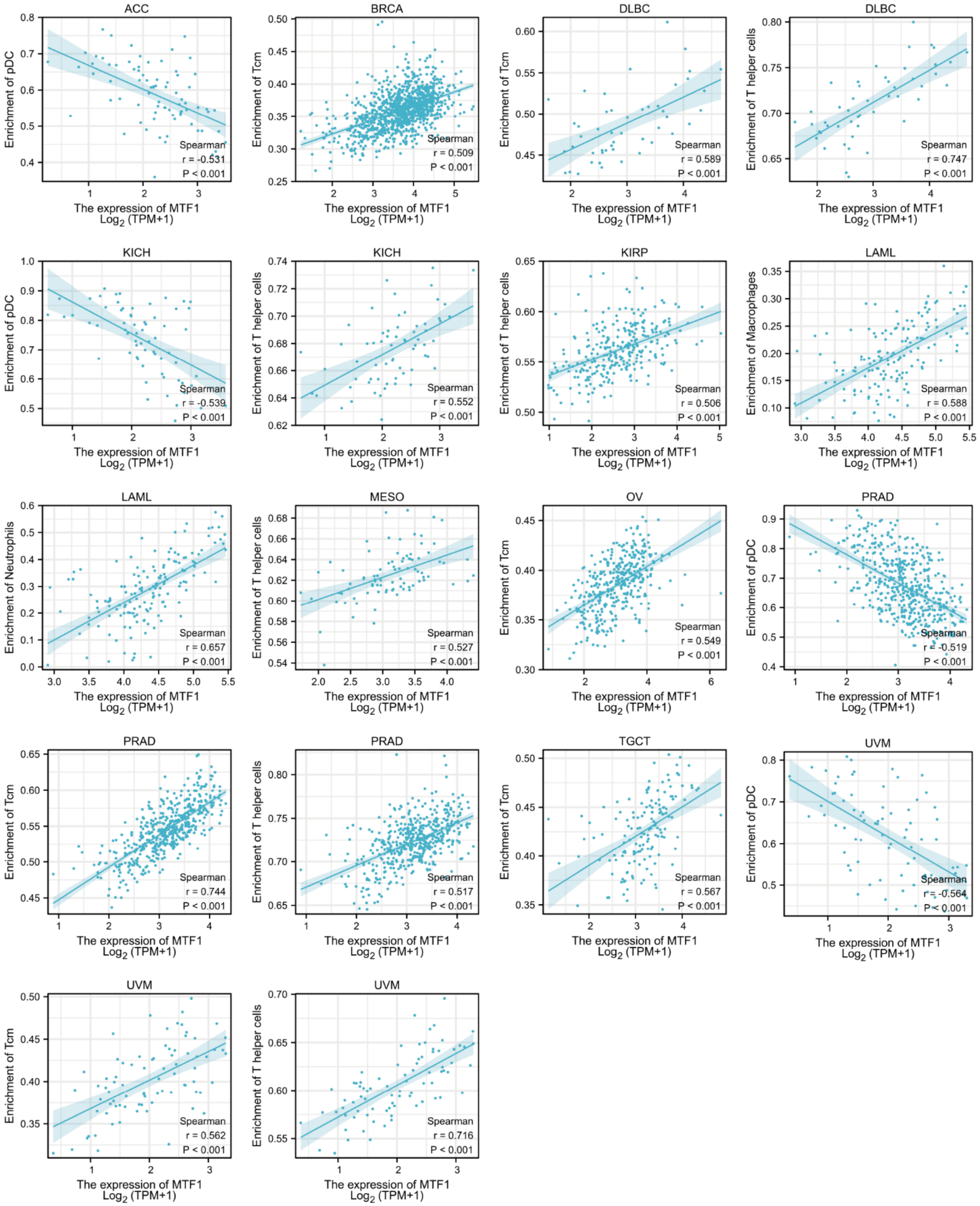

Supplementary Fig. S6. Correlation curves between MTF1 expression and immune cell infiltration.

Microscopic images of immunofluorescence were uploaded to this link:
https://www.jianguoyun.com/p/DR8dNMwQ0vfwChiMrNwEIAA .
